# RAIChU: automating the visualisation of natural product biosynthesis

**DOI:** 10.1186/s13321-024-00898-x

**Published:** 2024-09-03

**Authors:** Barbara R. Terlouw, Friederike Biermann, Sophie P. J. M. Vromans, Elham Zamani, Eric J. N. Helfrich, Marnix H. Medema

**Affiliations:** 1 Bioinformatics Group, Wageningen University, Droevendaalsesteeg 1, 6708 PB Wageningen, The Netherlands; 2 Institute for Molecular Bio Science, Goethe University Frankfurt, Max-von-Laue Strasse 9, 60438 Frankfurt am Main, Germany; 3 LOEWE Center for Translational Biodiversity Genomics (TBG), Senckenberganlage 25, 60325 Frankfurt am Main, Germany; 4 Senckenberg Gesellschaft für Naturforschung, Senckenberganlage 25, 60325 Frankfurt am Main, Germany

**Keywords:** Visualisation, Biosynthetic pathways, Natural product chemistry, Nonribosomal peptide synthetase, Polyketide synthase, Terpene, Ribosomally synthesised and posttranslationally modified peptide, Alkaloid

## Abstract

Natural products are molecules that fulfil a range of important ecological functions. Many natural products have been exploited for pharmaceutical and agricultural applications. In contrast to many other specialised metabolites, the products of modular nonribosomal peptide synthetase (NRPS) and polyketide synthase (PKS) systems can often (partially) be predicted from the DNA sequence of the biosynthetic gene clusters. This is because the biosynthetic pathways of NRPS and PKS systems adhere to consistent rulesets. These universal biosynthetic rules can be leveraged to generate biosynthetic models of biosynthetic pathways. While these principles have been largely deciphered, software that leverages these rules to automatically generate visualisations of biosynthetic models has not yet been developed. To enable high-quality automated visualisations of natural product biosynthetic pathways, we developed RAIChU (Reaction Analysis through Illustrating Chemical Units), which produces depictions of biosynthetic transformations of PKS, NRPS, and hybrid PKS/NRPS systems from predicted or experimentally verified module architectures and domain substrate specificities. RAIChU also boasts a library of functions to perform and visualise reactions and pathways whose specifics (e.g., regioselectivity, stereoselectivity) are still difficult to predict, including terpenes, ribosomally synthesised and posttranslationally modified peptides and alkaloids. Additionally, RAIChU includes 34 prevalent tailoring reactions to enable the visualisation of biosynthetic pathways of fully maturated natural products. RAIChU can be integrated into Python pipelines, allowing users to upload and edit results from antiSMASH, a widely used BGC detection and annotation tool, or to build biosynthetic PKS/NRPS systems from scratch. RAIChU’s cluster drawing correctness (100%) and drawing readability (97.66%) were validated on 5000 randomly generated PKS/NRPS systems, and on the MIBiG database. The automated visualisation of these pathways accelerates the generation of biosynthetic models, facilitates the analysis of large (meta-) genomic datasets and reduces human error. RAIChU is available at https://github.com/BTheDragonMaster/RAIChU and https://pypi.org/project/raichu.

**Scientific contribution**

RAIChU is the first software package capable of automating high-quality visualisations of natural product biosynthetic pathways. By leveraging universal biosynthetic rules, RAIChU enables the depiction of complex biosynthetic transformations for PKS, NRPS, ribosomally synthesised and posttranslationally modified peptide (RiPP), terpene and alkaloid systems, enhancing predictive and analytical capabilities. This innovation not only streamlines the creation of biosynthetic models, making the analysis of large genomic datasets more efficient and accurate, but also bridges a crucial gap in predicting and visualising the complexities of natural product biosynthesis.

**Supplementary Information:**

The online version contains supplementary material available at 10.1186/s13321-024-00898-x.

## Introduction

Natural products, also commonly referred to as specialised metabolites or secondary metabolites, are abundant in nature, and are produced by a range of microbes across different domains of life [1, 2]. They are structurally highly diverse and fulfil various important ecological functions [3–5]. Due to this wealth of structural and functional diversity, natural products have been exploited as drugs in human and veterinary medicine, as agrochemicals, and as molecular agents in the food and cosmetics industry [6]. Famous examples include the fungal nonribosomal peptide (NRP) antibiotic penicillin [7], the bacterial polyketide insecticide spinosad [8, 9], the polyketide immunosuppressant rapamycin [10], and the antibiotics daptomycin [11] and erythromycin [10], a NRP and a polyketide, respectively [1, 2].

Typically, microbial natural products are produced by enzymes encoded in biosynthetic gene clusters (BGCs): groups of genes that physically collocate to a genomic region and collectively encode the enzymes of a biosynthetic pathway. For this reason, BGC detection and annotation have inhabited a prominent role in natural product discovery pipelines. By predicting and visualising the chemistry encoded by a BGC, researchers can prioritise scaffolds with desirable properties from DNA sequence alone.

Broadly, BGCs can be subdivided into BGCs that encode modular assembly line-like biosynthetic pathways and BGCs that encode discrete multi-enzymatic assemblies [12].

NRPs and type I PKS-derived polyketides, are built by modular assembly line-like pathways implemented by non-ribosomal peptide synthetases (NRPSs) and polyketide synthases (PKSs), respectively. Core enzymes of textbook NRPSs and PKSs consist of multi-domain modules, each of which incorporates a peptide or polyketide building block (monomer) into the natural product scaffold in an assembly line-like fashion [3, 5]. In both systems, a minimal module comprises three domains: a non-catalytic carrier domain that functions as a thioester anchor for the building block that is incorporated by a given module; a recognition domain that selects the building block and covalently attaches it to the carrier domain via a thioester bond; and a synthesis domain, that covalently fuses the building block from the carrier domain to the growing natural product scaffold. In addition to these core domains, NRPS and PKS modules can also contain facultative enzymatic domains that modify building blocks, including the canonical β-carbon processing ketoreductase (KR), dehydratase (DH) and enoyl reductase (ER) domains in PKS systems. Terminal modules often contain an additional domain that releases the scaffold from the last carrier domain. In *trans*-AT PKS systems, *trans*-acting acyltransferase (AT) recognition domains are encoded by stand-alone genes within the BGC, *Trans*-AT polyketide scaffold predictions are based on association of the primary amino acid sequence of the ketosynthase (KS) synthesis domain with its substrate specificity [13]. The most commonly occurring PKS/NRPS domains and the reactions they catalyse are summarised in Figure S1 (Additional file 1). In textbook assembly line-like pathways, NRPS/*cis*-AT PKS module order and composition correspond with the order of monomers in the produced natural product and on-line modifications performed during assembly [5, 14]. This concept is known as the collinearity rule [5, 14]. Additionally, the arrangement of genes within BGCs often mirrors the order in which the corresponding enzymes operate during the biosynthesis of the associated NP [5, 14]. Structural diversity is further enhanced by accessory genes in NRPS and PKS BGCs, which encode enzymes that catalyse tailoring reactions [15, 16].

Still, bacteria and fungi also employ many assembly line independent strategies for specialised metabolism in the form of discrete multi-enzymatic assemblies. These include the production of RiPPs, terpenoids, and alkaloids. RiPP biosynthesis starts with the ribosomal translation of a gene-encoded precursor peptide, comprising a core peptide sequence flanked by leader and/or follower sequences. After ribosomal synthesis, precursor peptides undergo a series of post-translational modifications (PTMs), which are crucial for the structural and functional maturation of RiPPs [17]. Terpenoids are synthesised by terpene cyclases/synthases (TCs) from oligoprenyl precursors comprising isoprene units [18]. TCs group into two distinct types based on their catalytic mechanisms. Class I TCs activate the substrate by heterolytic cleavage of the terminal pyrophosphate group. The TC then generates the terpene hydrocarbon scaffold through a series of cationic cascade cyclization reactions, rearrangements, hydride and methyl shifts [18]. Class II TCs act through protonation of an olefinic (double-bonded) carbon in the substrate or an epoxide ring, leading to epoxide opening and kickstarting a set of carbo-cation cascade reactions in the opposite direction to generate terpene scaffolds [18]. Alkaloids are produced by three bioorthogonal strategies: NRPS-dependent, RiPP-dependent, and NRPS and RiPP-independent pathways. The latter of these constitute discrete multi-enzymatic assemblies, in which amino acids are modified by monofunctional enzymes to form the alkaloid product [19, 20].

As the catalytic domains of modular NRPS and PKS systems are well-characterised and building blocks selected by recognition domains can be predicted, it is possible to infer biosynthetic models and thus the core scaffold of the natural products associated with a BGC with reasonable accuracy. The same is the case for well-studied subclasses of RiPP and terpene biosynthetic pathways. While BGC detection platforms like antiSMASH and PRISM predict NP scaffolds, they do not supply detailed visualisations of the associated biosynthetic pathways [12, 21, 22]. Visual insight into the biosynthetic model and the natural product is desirable for multiple reasons: it gives an instant overview of the underlying biosynthetic chemistry to researchers not familiar with natural product biosynthesis, and it facilitates the identification of mistakes in structure predictions and links them to specific mispredicted biosynthetic reactions. However, manually drawing (predicted) biosynthetic models is a laborious and error-prone process. Automation of this will save researchers time and will reduce human error.

**Fig. 1:**
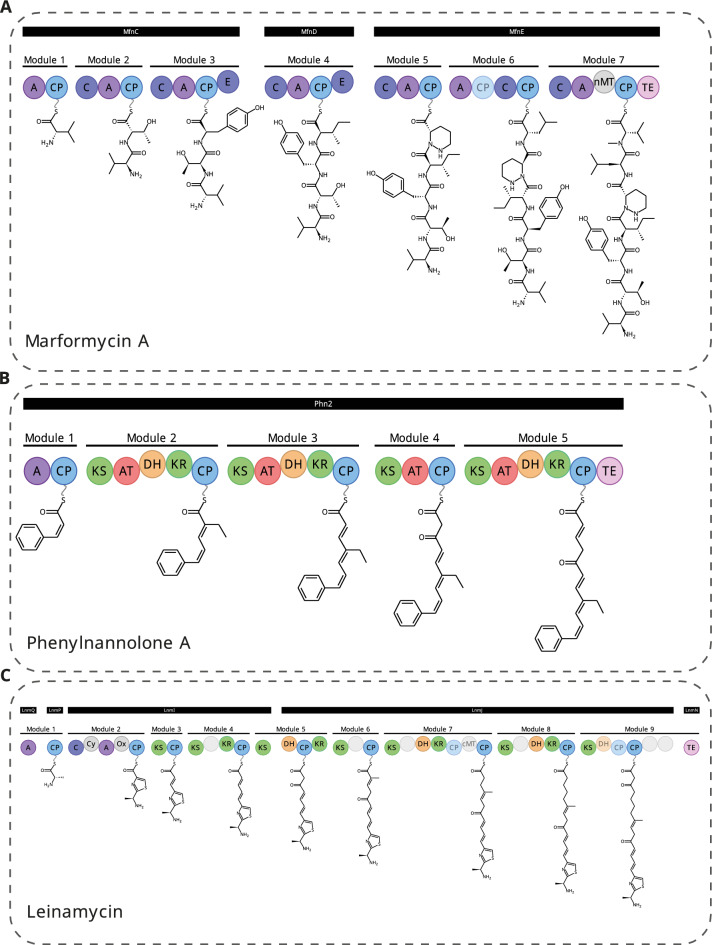
Examples of ‘spaghetti diagrams’ commonly used to depict biosynthetic pathways encoded by NRPS [25] (**A**), *cis*-AT PKS [26] (**B**), and NRPS-*trans*-AT PKS hybrid [27] (**C**) biosynthetic gene clusters. The above spaghetti diagrams were automatically generated by RAIChU

To enable automated, high-quality visualisation of biosynthetic models, we developed RAIChU: Reaction Analysis through Illustrating Chemical Units. Given a set of input domains/modules/enzymes and predicted or experimentally verified recognition domain specificities, RAIChU automatically produces ‘spaghetti diagrams’ (Fig. 1) depicting detailed biosynthetic models of PKS, NRPS, and hybrid PKS/NRPS pathways at module-resolution. Additionally, RAIChU provides custom biosynthetic pathway visualisations for RiPPs, terpenes and any other class of compounds for which predictions of enzymatic steps can be provided by the users. As such, RAIChU provides a solution for biologists, natural product chemists and bioinformaticians looking to automatically visualise biosynthetic pathways to facilitate the study of natural products.

## Results and discussion

To automatically visualise biosynthetic pathways, we created the software package RAIChU. RAIChU can visualise pathways of *cis*- and *trans*-AT PKS, NRPS, RiPP, alkaloid, and terpenoid biosynthesis and boasts a collection of tailoring enzyme reactions to decorate natural product scaffolds. RAIChU comprises two main libraries: the first is a reaction library that performs the *in-silico* chemistry required for scaffold assembly and tailoring; and the second renders visualisations of biosynthetic pathways as saveable images. For modular systems, these visualisations are spaghetti diagrams as described above (Fig. 1); for non-modular systems, they comprise standard reaction diagrams. Below, we discuss each of these libraries, assess RAIChU’s speed, and validate RAIChU on randomly generated and real-world examples.

### In-silico reactions

Biosynthetic assembly lines catalyse many biochemical transformations. To ensure the correctness of these transformations, we implemented the most commonly occurring elongation and tailoring reactions using PIKAChU, a tool which enforces chemical correctness of reaction intermediates by monitoring electron availability for the formation of bonds and lone pairs [23]. This approach is different from the one taken by antiSMASH, which relies on SMILES [24] concatenation to compute putative products, at times leading to the presence of extra atoms at both termini of linear molecules [22]. Similar to PRISM, we opted for a graph-based approach, which better represents the underlying chemistry and enables the implementation of reactions that are difficult to achieve through linear SMILES concatenation, like cyclisations [21].

### Modular systems

For assembly line-like pathways like NRPSs/PKSs, reactions are determined and computationally executed per module based on module composition and substrate specificity of enzymatic domains. These reactions involve electron redistribution to break and form bonds. For both NRPS and PKS systems, we defined four module types: starter modules, elongation modules, non-elongating modules (*trans*-AT PKS), and termination modules. In RAIChU, every cluster should be defined with exactly one starter module, followed by any number of elongation modules, and optionally completed with one termination module. NRPS and PKS starter modules minimally require a recognition (A/AT) domain and a carrier (PCP/ACP) domain. Elongation modules additionally require a condensation (C/KS) domain, and termination modules are elongation modules with a terminal TE, C or TD domain. *Trans*-AT PKS modules do not require a recognition (AT) domain, but minimally require an ACP domain for starter modules, and a KS and ACP domain for elongation and termination modules (Table 1).

**Table 1:** Minimal domain requirements for RAIChU modules

	*cis-*AT PKS	*trans-*AT PKS	NRPS
Starter module	AT-ACP/CAL-ACP	ACP	A-PCP/CAL-PCP
Elongation module	KS-AT-ACP	KS-ACP	C-A-PCP/Cyc-A-PCP
Termination module	KS-AT-ACP	KS-ACP	C-A-PCP/Cyc-A-PCP

Requirements depend on module type and position. In lenient mode, a module is labelled as broken if it does not minimally contain the indicated domains. In strict mode, broken modules raise an error. Termination modules may also contain thioesterase (TE) or terminal reductase (TD) domains

Additionally, each module can contain modifying domains, each corresponding to a tailoring reaction executed by RAIChU. NRPS modules can contain E, nMT, Cyc and Ox tailoring domains, executing epimerisation, N-methylation, cyclisation, and oxidation, respectively. PKS modules can contain KR, DH, and ER β-carbon processing domains, catalysing ketoreduction, dehydration and enoyl reduction (Figure S1); α-carbon methyltransferase domains catalysing methylation; and β-branching components involved in β-carbon exo/endo methylene formation. Any domain can be set to ‘inactive’, such that the domain is ignored during scaffold assembly. Within a module, only the first active domain of a domain type is considered active; any subsequent domains of the same type are automatically set to inactive.

In *trans*-AT PKSs, transformations are predicted from the substrate specificity of the downstream KS domain, not by the presence/absence of domains in the PKS itself. For this reason, transformations are selected and automatically performed by RAIChU by assigning one of the 44 KS domain subtypes that antiSMASH can predict [22]. *Trans*-AT PKS modification reactions are internally executed by ‘dummy domains’, which are determined by the KS domain subtype and are not visualised in RAIChU’s biosynthetic diagrams. Additional biosynthetic domains can be visualized by the user by manually adding them to the diagram as ‘unknown’ domains with user-defined names of three letters or less. Importantly, these domains are not used by RAIChU internally to compute the chemistry.

In all modular systems, reactions are only executed if the reaction is chemically possible; otherwise, the domain executing the reaction is automatically set to ‘inactive’.

In addition to module architecture, RAIChU also requires the substrate specificity of each active recognition domain. To ensure compatibility between RAIChU and antiSMASH [22], we selected those substrates that antiSMASH [22] can predict, in addition to substrates that occurred in the training sets of the A domain specificity predictors AdenylPred [28], NRPSPredictor2 [29], SANDPUMA [30], and PARAS/PARASECT (Terlouw et al., in preparation). For A domains in NRPS elongation and termination modules, RAIChU supports 198 substrates: the 20 proteinogenic amino acids, 169 non-proteinogenic amino acids, including 12 d-amino acids, 8 β-amino acids, and 1 wildcard, for which the amino acid side chain constitutes a rest group. NRPS starter modules can incorporate an additional 79 carboxylic acid substrates, and termination modules an additional 4, for a total of 281 NRPS substrates. For AT domains in PKS elongation and termination modules, there is a choice of 5 malonyl-CoA derivatives and 1 wildcard. PKS starter modules support a wider variety of 25 chemically diverse substrates.

**Fig. 2:**
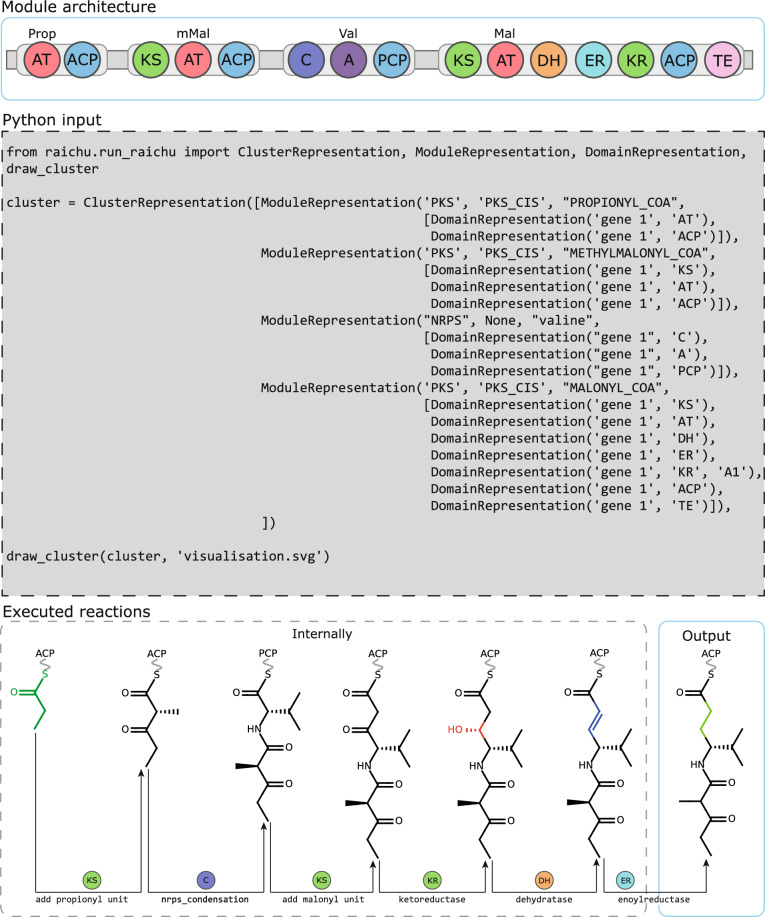
A biosynthetic pathway simulated by RAIChU. The hybrid NRPS/PKS gene depicted at the top is processed one module at the time. C and KS domains catalyse elongation reactions, extending the scaffold with the subunit recognised by A and AT domains respectively. On-line modification reactions are processed after elongation reactions. Internally, RAIChU processes cluster architectures as cluster representation objects (grey box)

One module at a time, RAIChU executes elongation and on-line modification reactions catalysed by facultative enzymatic domains within the module on the nascent chain. First, the elongation reaction catalysed by the C or KS domain is executed, and the resulting product is covalently tethered to the module’s carrier domain. Then, all modifying reactions are performed (Fig. 2). When a TE, C or TD domain is encountered in a terminal module, the reaction product is detached from the carrier domain through a hydrolysis reaction. Finally, potential sites of macrocyclization are determined, and candidate macrolactone and macrolactam products as well as the linear product are computed.

We sought to improve upon existing drawing software used by antiSMASH [22] and PRISM [21] to visualise predicted NRP and polyketide products by incorporating stereochemical information. This addition is especially relevant for the α-carbons of amino acid building blocks and is often dictated by the presence/absence of E domains. The same holds true for the chiral polyketide intermediates generated by ketoreductases, which depend on the subtype of KR domains [31]. When an E domain is present in a module, RAIChU performs epimerization on the α-carbon of l-amino acid substrates. We also implemented the KR domain subtypes A1, A2, B1, and B2, which each yield different polyketide stereoisomers; subtype C1, which is inactive; and subtype C2, which has no ketoreductase activity but does catalyse epimerisation (Figure S2) [31].

### Discrete multi-enzymatic assemblies

Another class of natural products for which the scaffold structure can be predicted by current tools are RiPPs [12, 17, 32]. RAIChU computes RiPP structures in three steps: first, it constructs an initial scaffold from the amino acid sequence of the precursor peptide; second, RAIChU tailors the scaffold with user-specified reactions representing family-specific post-translational modifications; and third, it cleaves off leader and/or follower peptides by hydrolysing the corresponding peptide bonds.

RAIChU also supports a library for automating the visualisation of terpenoid and alkaloid biosynthesis. Currently, this would be based on manually hypothesised or experimentally verified biosynthetic mechanisms, but in the future, in silico predictions may become available for these natural product classes as well. RAIChU builds terpenoid backbones by allowing the user to pick one of 6 oligoprenyl precursors. The user then defines the terpene cyclase type (class I or II), cyclisation sites (the carbon atoms between which a cycle-forming bond is created), double bond isomerizations, and methyl shifts to cyclise the precursor. When a type I terpene cyclase is selected, the diphosphate is removed from the product in the process. Note that we did not intend to visualise the exact reactions taking place during the carbo-cationic cascade reactions. Instead, we aimed to provide the user with reaction ‘shortcuts’ that can be used to visualise the correct product of a terpene cyclase. The resulting hydrocarbon backbone can subsequently be decorated by a range of tailoring reactions described below.

Additionally, RiPP- and NRPS-independent alkaloid pathways can be implemented in RAIChU by using an amino acid as an initial scaffold, which can subsequently be processed by an array of tailoring reactions.

### Tailoring reactions

**Fig. 3:**
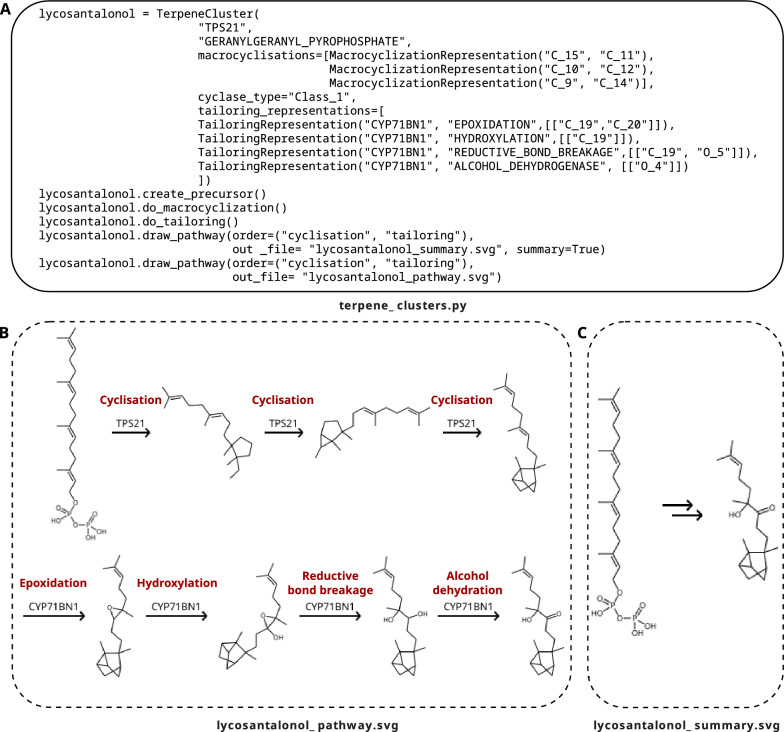
Automated visualisation of the reaction pathway of the plant terpene lycosantalonol [34] as implemented in RAIChU. Implemented code is shown in (**A**). As some reactions, such as cationic cascade reactions during terpene biosynthesis, are difficult to program in a standardised manner, RAIChU provides the option to visualise the pathway as it was implemented (**B**) as well as a summarised pathway which only shows the first and last reaction intermediate (**C**). Note that RAIChU places all structures in the pathway next to one another from left to right, rather than visualising them across multiple rows as shown in C. However, individual elements of the SVG image are easily movable in image editors for publication purposes. For enhanced clarity and ease of understanding, red labels have been manually incorporated into the figure

**Fig. 4:**
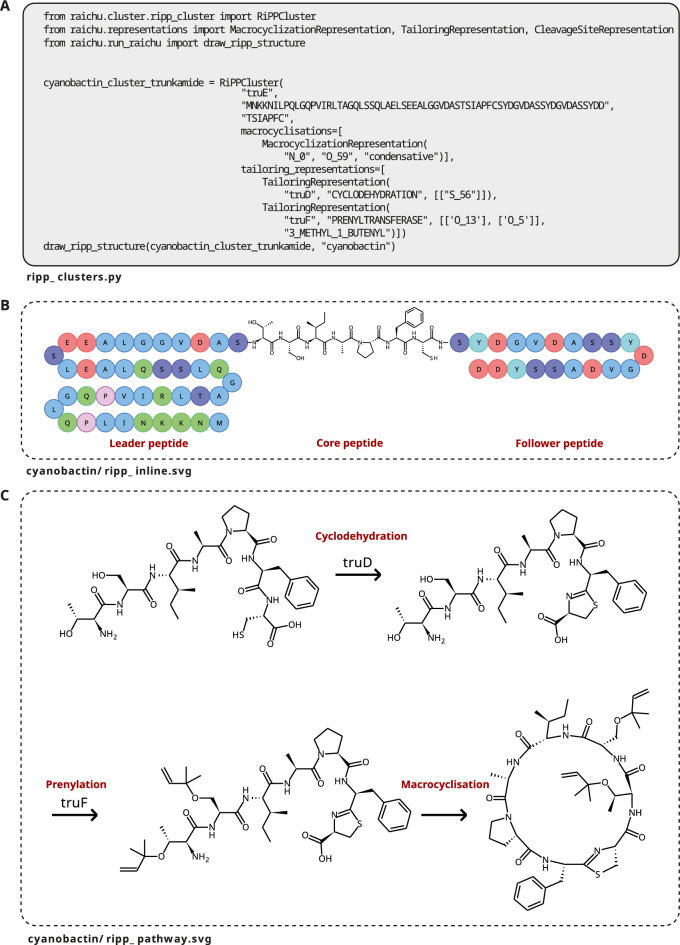
Automatic visualisation of the biosynthetic model for the RiPP-derived trunkamide [35] as implemented in RAIChU. Code is shown in (**A**), core peptide visualisation in (**B**), and tailoring modifications and cyclisations in (**C**). For enhanced clarity and ease of understanding, red labels have been manually incorporated into the figure

Most natural products are modified by tailoring reactions after initial scaffold assembly [33]. To visualise this important step in scaffold diversification, we implemented 34 tailoring reactions (Table S1; Additional file 1) that can be called within the context of class-specific cluster frameworks for both assembly line-like pathways and discrete multi-enzymatic assemblies (Figs. 3, 4). These reactions include the family-specific posttranslational modifications of 25 RiPP families, enabling advanced RiPP chemistry. As it is currently not possible to predict the regio and stereochemistry of most of these tailoring enzymes, users need to specify their reaction targets. To this end, RAIChU provides a function to automatically detect and visually highlight all atoms that specific tailoring reactions can be performed on (see RAIChU wiki at https://github.com/BTheDragonMaster/RAIChU/wiki).

In modular systems, off-line tailoring enzymes perform reactions after the NRP/PK core has been released from the assembly line. In RiPP and terpene systems, tailoring, cyclisation and/or proteolytic cleavage reactions are performed in any desired order, as long as the reactions within each category are performed sequentially. If a different reaction order is desired, for instance if cyclisations need to be performed both before and after tailoring, the PROTEASE or MACROLACTAM_FORMATION tailoring enzymes can be used instead of the default built-in proteolytic cleavage or cyclisation functions. Given any set of reactions and reaction targets, RAIChU can automatically visualise the resulting biosynthetic model (Figs. 3, 4).

As many reactions, such as cationic cascade reactions in terpenes, are difficult to program in a standardised manner, RAIChU takes various programmatic shortcuts to perform these reactions. Consequently, it is possible that some reaction intermediates in RAIChU may not represent a true reaction intermediate in nature. For this reason, RAIChU also provides the option to display a summarised reaction pathway, which only displays the first and last reaction intermediate (Fig. 3C).

### Automated biosynthetic model visualisation of modular systems

After performing chemical reactions according to a BGC’s encoded module architecture, RAIChU produces two visual outputs: a spaghetti diagram depicting the complete biosynthetic pathway of a natural product at module resolution (Figure S3A; Additional file 1), and images of all possible linear and cyclic products (Figure S3B; Additional file 1). Biosynthetic domains can be assigned to the genes encoding them, which show up as labels above the spaghetti diagram (Figure S3A; Additional file 1). RAIChU also supports the visualisation of modules that are split across multiple proteins (Fig. 1C).

To produce a large number of cluster images at once, it is possible to script RAIChU in Python. Users are provided with three options: the user can load antiSMASH output in .gbk format and produce the desired output (Figure S4A; Additional file 1); the user can directly script module architectures into Python as cluster representations, which can be interpreted by RAIChU (Fig. 2, grey box); or the user can provide a tab-separated text file containing the gene and module architecture of custom clusters, an example of which can be found under raichu/example_tab_separated_input_file.txt (Figure S4B; Table S2; Additional file 1). With a single function call (Figure S4C; Additional file 1), the user can then compute the product(s) of the  (Figure S4D; Additional file 1). Independent of the data format chosen, the user can choose to render spaghetti diagrams, reaction products, or both. SMILES strings of reaction products can also be stored.

### Validation of RAIChU on modular cluster architectures

To validate RAIChU’s visualisation accuracy and readability, we randomly generated 1000 NRPS BGCs, 1000 *cis*-AT PKS BGCs, 1000 *trans*-AT PKS BGCs, 1000 hybrid *cis*-AT PKS/*trans*-AT PKS BGCs and 1000 hybrid NRPS/PKS BGCs, randomly selecting the number of modules per cluster, module type (NRPS or PKS), domains within modules, substrate specificities of A and AT domains, and KR domain subtype such that the rules we previously laid out for NRPS and PKS cluster and module architectures were obeyed. We manually assessed each spaghetti diagram on three characteristics: the correctness of the depicted structures; the angle of bonds attached to the backbone, which should be 0° or 180° with respect to the drawing plane; and overlap of functional groups with each other and with other drawing elements such as gene, module, and domain depictions. We achieved 97.62% readability and 100% accuracy on all randomly generated sets of clusters (Figure S5 and Tables S4, S5; Additional file 1). Spaghetti diagrams are available as separate.svg files, and.gif files, showing an animation of all generated clusters per validation set (10.5281/zenodo.10987931).

To assess its ability to automatically generate visualizations of NRPS and PKS biosynthetic assembly lines, we additionally tested RAIChU on antiSMASH predictions of real-world BGCs from the MIBiG database: a database of experimentally verified biosynthetic gene clusters for which both DNA sequence and the associated natural product are known [36] (Table S3; Additional file 1). First, we ran RAIChU on the entire database to ensure no unexpected errors occurred. For each cluster, we produced both spaghetti diagrams and visualised predicted products that arise from tail-to-scaffold cyclisations. We only encountered two types of errors, both of which were expected: they occur when running RAIChU on clusters with no modules or clusters with no complete modules.

**Fig. 5:**
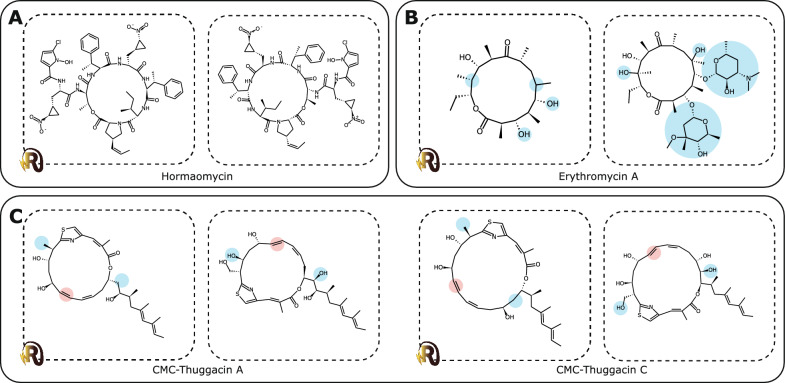
Examples contrasting predicted products visualised by RAIChU (left) with experimentally characterised products (right). Inconsistencies due to lacking stereochemical information (red) and unpredicted tailoring modifications (blue) are indicated with coloured circles

Next, in order to give users an impression of how well RAIChU models reflect biosynthetic reality, we tested the structural accuracy of RAIChU’s scaffold visualisations based on either antiSMASH predictions or MIBiG annotations on a subset of 10 *cis*-AT PKS clusters, 8 *trans*-AT PKS clusters, 10 NRPS clusters, and 10 NRPS/PKS hybrid clusters (Table S3; Additional file 1). We found that RAIChU’s spaghetti diagrams matched with published core scaffold biosynthesis models, and among the computed products there was often at least one that was very similar to or identical to the experimentally characterised product (Fig. 5; Figures S6-S9, Additional file 1). Discrepancies usually arose for one of three reasons: post-assembly tailoring reactions, such as the glycosylations in erythromycin [10] (Fig. 5B)—RAIChU can execute these reactions, but no current tools can predict their regioselectivity; double bond stereochemistry following dehydration reactions in PKS systems, as observed in the CMC-thuggacins (Fig. 5C); and absence of a required substrate in RAIChU, such as the fatty acid starter substrates incorporated into lipopeptides [37] (Figure S8; Additional file 1). The latter can be easily solved by programmers by adding the desired substrate to RAIChU manually (see RAIChU wiki).

RAIChU’s visualisation of predicted products worked especially well for NRPS BGCs (Fig. 5A), which produce relatively unreactive scaffolds compared to polyketide products which often contain many accessible hydroxy- and keto-groups. Additionally, NRPS products often undergo fewer post-assembly modifications than PKS products. Also, their module architectures are usually comparatively simple.

Despite these shortcomings, scaffold assembly is highly accurate both in terms of stereochemistry and structure, with stereocenters always inferred correctly given correct annotation of KR domain subtypes and epimerisation domains. For some BGCs that are involved in the production of multiple natural products, RAIChU was able to generate structures corresponding to each of them, such as the CMC-thuggacins which are produced from one BGC (Fig. 5C). Finally, with RAIChU’s diagrams we were able to identify that the colistin A BGC in the MIBiG database [36] points to the wrong PubChem entry, as this cluster likely produces an analogue of colistin A with a d-2,4-diaminobutyric acid residue at position 3 instead of an l-2,4-diaminobutyric acid, as also postulated by the authors who originally published the BGC [38]. This example demonstrates the need for automatic visualisation to guide researchers when they make chemical diagrams to minimise human error.

### Speed assessment

**Fig. 6:**
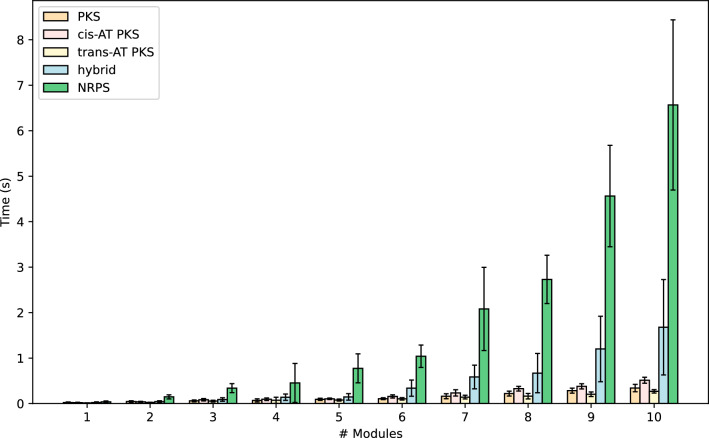
Speed assessment of RAIChU tested on randomly generated biosynthetic gene clusters. Average speed and standard deviation were based on 10 BGCs for each category

We measured RAIChU’s speed by randomly generating spaghetti diagrams for 1–10 module BGCs, generating 10 BGCs per module number (1–10) and module type (PKS, *trans*-AT PKS, *cis*-AT PKS, NRPS, and hybrid). RAIChU performed fastest for PKS clusters, with an average drawing time of 2.73 s per cluster. Drawing NRPS and NRPS/PKS hybrid clusters takes slightly longer: 9.62 s and 5.21 s per cluster, respectively. This is likely due to the larger size of NRPS side chains, which require more overlap resolution steps to avoid clashes. Computing time increased with module number (Fig. 6). Drawing time increases exponentially with module number.

### Accessibility and future challenges

RAIChU can be installed as a Python pip package for scripting purposes (https://pypi.org/project/raichu/). This implementation makes RAIChU easily available for programmers looking to automate modular NRPS/PKS visualisation as a part of larger BGC prediction pipelines, researchers who want to assess the putative biosynthetic model for a cluster they have found, database maintainers who want to cross-reference compounds with BGCs to record the substrate specificities of recognition domains, and bioengineers who want to explore how existing BGCs can be edited to produce novel compounds. With an average cluster rendering time of under 6 seconds, RAIChU is also suitable for large-scale automatic rendering of biosynthetic pathways and NRP/polyketide product scaffolds.

Currently, one of RAIChU’s main shortcomings is the requirement for providing reaction targets for post-assembly tailoring reactions, which makes it challenging to automatically script complete biosynthetic models using RAIChU. One option would be to take a combinatorial approach as seen in PRISM, which performs all possible tailoring reactions on all possible reaction targets yielding often large libraries of putative structures [21]. As RAIChU is a visualisation tool rather than a prediction tool, we envision that the incorporation of automatic reaction site detection will be added to RAIChU as the community develops better tools to predict the stereo- and regiospecificity of tailoring enzymes, a process that RAIChU can hopefully play a part in by facilitating the study of novel natural product BGCs.

For now, RAIChU’s usability is limited by the chemistry that the community is able to predict with existing tools. As such, future usability will rely on the continuous development of software that can improve the prediction of natural product biosynthetic pathways, such as the MITE initiative (manuscript in preprint [39]). Likewise, RAIChU’s added value in bioengineering efforts will rely on external tools and technologies such as the NRPS/PKS cluster engineering aid ClusterCAD [40], and NRPS exchange unit technology [41] that can predict which NRPS domains and enzymes are biosynthetically compatible. RAIChU’s underlying software architecture, which includes continuous integration testing and a modular code base, is set up such that future changes and integration in larger software suites can easily be implemented by the community.

Ultimately, we plan to integrate RAIChU into antiSMASH and MIBiG to directly couple BGC prediction to visualisation, and to provide biosynthetic models for newly deposited entries, respectively.

## Conclusion

We developed RAIChU: a novel tool for rapid automatic visualisation of natural product biosynthesis. With options to render predicted, hypothesised or experimentally verified biosynthetic pathway visualisations for diverse natural product biosynthetic classes, RAIChU can produce valuable visual output that can guide researchers within and outside the natural product field to better understand novel BGCs they encounter. RAIChU is prepared for future deployment by supporting a library of biosynthetic reactions that are currently difficult to predict in silico, but can be used to visualise manually hypothesised reactions and pathways, or can be coupled to novel predictive tools once they become available. Additionally, RAIChU lets researchers encode and visualise biosynthetic pathways that were not predicted from the associated BGCs but instead were experimentally characterised. As such, RAIChU provides a solution for researchers and programmers looking to quickly obtain visual overviews of predicted and characterised natural product biosynthetic pathways.

## Methods

### Software description

RAIChU (v1.0.2) is implemented in Python (v3.9.12). It utilises three external Python dependencies: matplotlib [42] (v3.5.0), PIKAChU [23] (v1.1.1) and BioPython [43] (v1.83).

### Reaction implementation

#### Scaffold assembly of modular systems

Assembling a modular scaffold starts with defining the core and tailoring enzymes encoded by a biosynthetic gene cluster. For this purpose, RAIChU employs a module for storing representations of biosynthetic units, including clusters, modules, domains, and tailoring enzymes. These representations can subsequently be converted into ModularCluster objects, which contain methods for structure computation and diagram visualisation. Similarly, ModuleRepresentations are converted into Module objects, and DomainRepresentations into Domain objects.

Clusters can be built from cluster representations in strict mode or lenient mode. In strict mode, the cluster can only be built if all domains and substrates are recognised by RAIChU, and if modules contain all necessary domains to be functional. In contrast, lenient mode allows for broken modules, the inclusion of unrecognised domains as ‘unknown’ domains, and the incorporation of unknown substrates as wildcards.

A module is considered complete if it contains all necessary domains for biosynthetic chain elongation (with the exception of some *trans-*AT PKS modules, which can be non-elongating). In lenient mode, incomplete modules are labelled as broken, and are not processed during scaffold assembly. Processable assembly line architectures start with a starter module and end with a termination module, with any number of elongation modules in between. Domain requirements are summarised in Table 1.

After building a cluster from its representation, various methods can be called to execute sequential biosynthetic steps, generate pathway diagrams, and compute putative products. Biosynthetic reactions are executed one module at a time, with Module objects organising the reactions and Domain objects executing them. As many domain types catalyse similar reactions, reaction functions are stored in globally accessible modules.

An elongation cycle starts by processing the to-be-incorporated building block, which is stored as a Module attribute. To this purpose, we first convert SMILES strings of NRPS and PKS building blocks to graph-based structure objects using the PIKAChU cheminformatics library [23]. RAIChU only permits valid SMILES describing the following molecules: any carboxylic acid for NRPS starter modules; any amino acid or β-amino acid for NRPS elongation and termination modules; any thioester for PKS starter modules; and built-in SMILES representing malonyl-CoA, methylmalonyl-CoA, ethylmalonyl-CoA, methoxymalonyl-ACP, or a wildcard extender unit for PKS elongation and termination modules.

Once the SMILES string has been processed, the starter unit is covalently bonded via a thioester to an ACP or PCP Domain instance: a RAIChU object that largely behaves as a PIKAChU Atom object. This implementation mimics the transfer of the substrate onto the phosphopantetheinyl arm of an ACP/PCP domain. For NRPS starters, this transfer is executed through PIKAChU’s built-in condensation function. For PKS starters, the subunit is transferred from the CoA sulphur atom to the sulphur atom attached to the carrier domain via transesterification (Figure S1, thiolation reaction and acyltransferase reaction).

For NRPS elongation reactions, two reaction targets are identified with PIKAChU’s substructure search function: a thioester within the nascent peptide that is attached to the upstream module’s PCP; and the nitrogen atom of an amino acid or β-amino acid backbone in the new building block. Next, using PIKAChU’s built-in hydrolysis and condensation functions, the thioester is hydrolysed to release the previous chain intermediate from the carrier domain, and the released scaffold is covalently linked to the new building block through a condensation reaction to form a new peptide bond. Finally, the nascent peptide is tethered to the PCP domain through another transesterification reaction (Figure S1, condensation reaction).

For PKS elongation reactions, we employed SMILES [24] that represent truncated malonyl-CoA derivatives that lack their terminal carboxyl group, as the ketosynthase reaction decarboxylates the malonyl-CoA derivative. A substructure search identifies the thioester bond linking the nascent polyketide to the upstream ACP domain, and the thioester bond is hydrolysed to release the chain intermediate. The thioester carbon is then covalently linked to the downstream ACP domain via transesterification, and a bond is formed between the ⍺-carbon of the new building block and the former thioester carbon of the nascent chain. This reaction extends the chain intermediate and attaches it to a carrier domain (Figure S1, ketosynthase reaction).

#### Scaffold release from modular systems

In modular systems, the nascent chain is released from the carrier domain and returned as a product once all elongation and modifications installed by facultative enzymatic domains have been performed in the first-encountered termination module. RAIChU supports two options for chain release: linear and circular. For NRPS and PKS systems, RAIChU provides the option to automatically cyclise the released scaffold. For this purpose, it assesses the presence of intramolecular hydroxyl and amino groups that could be used by the TE/TD/C domain to release the product by means of cyclization, generating lactones or lactams, respectively. All resulting circular products are returned as PIKAChU Structure objects.

#### Scaffold assembly of discrete multi-enzymatic assemblies

Similar to modular systems, RiPP, terpene and alkaloid clusters are stored in Cluster objects which contain all necessary methods for computation and visualisation.

For RiPPs, the scaffold is assembled from the primary amino acid sequence of the core peptide. In contrast to NRPs, RiPPs contain solely proteinogenic amino acids prior to PTMs and are synthesised in one step. Therefore, peptide backbone generation can be sped up substantially by concatenating the SMILES strings for all individual amino acids and converting the final SMILES to a PIKAChU structure object. Alternatively, the complete precursor can be loaded as a PIKACHU structure object and subsequently cleaved proteolytically.

Alkaloid scaffold assembly utilises a single amino acid as a starter, parsed as a PIKAChU structure object from a SMILES string. This starter can be subsequently modified by tailoring enzymes.

Terpene scaffolds are assembled from a SMILES string representing an oligoprenyl precursor, and parsed into a PIKAChU structure object. Subsequently, the action of the terpene cyclase is simulated by a combination of three reactions:

1. Double-bond isomerisations
2. Methyl-shifts
3. Macrocyclisations

Double bond isomerisations are performed by reducing the original double bond and then oxidising a specified adjacent single bond. Methyl-shifts are employed by performing reductive bond breakage between the to-be-transferred carbon and the adjacent carbon, followed by addition of a methyl group to the target carbon.

For Class II TCs, the precursor is dephosphorylated. Macrocyclisations are implemented as an oxidative bond formation, where the bonds between target carbons and their adjacent hydrogen atoms are broken and subsequently a bond between the two carbon atoms is built.

#### Determination of polyketide/peptide backbones

For generating readable spaghetti diagrams, it is key that the polyketide/peptide backbone is drawn vertically (Fig. 1). The same challenge applies to horizontally drawn RiPPs and their abbreviated leader and precursor peptides, and to substrates recognised by the starter modules of NRPs and polyketides, which can be quite large and need to be drawn as vertical as possible for visualisation purposes. Therefore, RAIChU employs various different strategies for finding the atoms in polyketide/NRP building blocks and RiPPs that constitute the backbone in the final product: one for polyketides; one for amino acids, β-amino acids, and acids; and one for the polypeptide backbone of RiPPs. In all cases, atoms belonging to the central chain are flagged with PIKAChU annotations, which can later be used by RAIChU’s drawing algorithm to correctly draw the central chain.

Identification of the central chain for amino acids, polypeptide backbones, and β-amino acids is accomplished through PIKAChU substructure searches. Cyclic β-amino acids where three backbone atoms partake in the cycle are treated as carboxylic acid NRP starter units (see below). The central chains of starter amino acids and β-amino acids that contain a chain of six or more carbons in the side chain are also determined using the function for carboxylic acid NRP starters. Central chain identification for polyketide starter and extender units was hardcoded for each starter/elongation unit supported by RAIChU.

It can occur that reactions catalysed by facultative enzymatic domains in NRPS and *trans*-AT PKS systems create microcycles on the scaffold. When this cycle contains more than three central chain atoms, RAIChU attempts to find an alternative shorter path through the cycle. When a path containing only two or three backbone atoms is found, the central chain is changed to include the atoms in this alternative path.

We defined the central chain of carboxylic acid starter units as the longest chain of noncyclic atoms. When a ring is encountered in the central chain, as is the case for substrates like hydrocinnamic acid and 2-carboxyquinoxaline, RAIChU calculates the subtree sizes of the cycle’s non-cyclic outgoing bonds, considering only paths that need one, two or three atoms to traverse the cycle to reach the outgoing bond. If such bonds are found, the bond leading to the largest subtree is selected, the atoms that make up the shortest path to this outgoing bond are labelled as central chain atoms, and the chain is continued linearly in the largest subtree. If no suitable outgoing bonds are found, the central chain extends three atoms into the ring and then terminates to ensure good drawing results.

#### Tailoring reactions

The tailoring reactions implemented into RAIChU fall into five subgroups: group transfers, eliminations, oxidoreductions, epimerisations, and cyclisations. They can be deployed as either on-line tailoring reactions that are typically catalysed by facultative enzymatic domains within the NRPS/PKS enzymes of modular systems; and off-line tailoring reactions that are catalysed by enzymes encoded by stand-alone genes. The reaction order of modifications catalysed by facultative enzymatic domains such as the three canonical β-carbon processing domains in PKS systems (KR, DH, and ER domains) is hard-coded according to known biosynthetic rules, while the reaction order of post-assembly reactions is defined by the user.

Tailoring reactions will only be performed if the reaction is chemically possible on the target structure. As such, on-line tailoring reaction functions always return a tuple: the (un)tailored structure, and a boolean expression indicating whether the reaction was successfully performed or not. If a post-assembly tailoring functions instead raise an error if the reaction cannot be performed. A comprehensive list of RAIChU’s tailoring reactions can be found on the RAIChU wiki.

In group transfer reactions such as *N*-methylations in NRPS systems (Figure S1: *N*-methylation), RAIChU first breaks the bond between the target atom to which the group is to be appended and an adjacent hydrogen atom. It also removes a hydrogen atom from the transferred group. Subsequently, a new bond is formed between the group and the target atom. Elimination reactions, such as the reactions performed by arginases or spliceases, are encoded in reverse: first, RAIChU breaks the bond adjacent to the group that is to be removed and then adds hydrogen atoms to both the removed group and the scaffold.

Oxidoreductions deal with the formation, reduction, and shifts of double and/or aromatic bonds. These reactions typically involve adding or removing hydrogen atoms and changing the hybridisation state of atoms adjacent to the modified bonds. Examples are the four stereo-specific ketoreductions performed by KR domains (Figure S1: ketoreduction; Figure S3) and double bond shifts performed by isomerases. These reactions are implemented using various PIKAChU methods, including two functions that change bond order.

In epimerisation reactions, the α-carbon of a RiPP or NRP amino acid building block is identified through a substructure search. The chirality flag of this atom is then reversed to invert the stereochemistry (Figure S1, epimerisation reaction).

To implement cyclisation reactions, RAIChU forms a new bond between two non-adjacent target atoms. Examples are two NRPS chain release mechanisms: reductive cyclisation catalysed by the TD domain and macrolactam/macrolactone formation by the TE or C domain.

### Visualisation

RAIChU draws two types of images for assembly line-like pathways: spaghetti diagrams and fully mature products. For discrete multi-enzymatic assemblies, the precursor and the cyclized, tailored, and final products are visualised. RAIChU relies on PIKAChU’s drawing engine for all diagram types, but requires an important addition for spaghetti diagrams: the central chains of the molecules need to be positioned vertically (or horizontally for RiPPs), such that all bonds in the central chain sit at alternating angles of 60° and -60° with respect to the drawing plane, with the exception of bonds involved in the formation of a cyclic structure, which sit at a 90° angle such that the polygon of the side chain can protrude symmetrically from the central chain (Fig. 1A, module 5). For this purpose, we created a RaichuDrawer class which inherits from PIKAChU’s Drawer class. It first positions atoms with PIKAChU’s drawing algorithm, then adjusts the angles of the molecule’s central chain, placing the carrier domain at the top, the attached sulphur atom of the thioester directly beneath it, and the carbon of the thioester at an angle of 60° below it. Next, the rest of the atoms of the central chain are placed at alternating angles of − 60° and 60°, rotating side chains to sit at 0° or 180° with respect to the drawing plane. For bonds involved in the formation of a cyclic structure in the central chain, the entire cycle is mirrored if it sits on the wrong side of the bond, positioning it left of the bond if the previous angle was 60°, and right otherwise. Finally, RAIChU resolves steric clashes using PIKAChU’s secondary overlap resolution function and a custom finetuning function which prevents the rotation of bonds in or attached to the central chain whilst finding the optimal angle for other bonds in the structure to minimise clashes.

Spaghetti diagrams are encoded in scalable vector graphic (SVG) format. In spaghetti diagrams, domains are represented as circles and genes as rectangles. Structures are aligned with the circles representing carrier domains by moving each atom in the structure by the same translational operation such that the coordinates of the domain instance in the structure match those of the circle representing the module’s carrier domain. The module architecture and all structures are then rendered to the canvas and saved in SVG format. Biosynthetic products are drawn using PIKAChU’s drawing library and saved in SVG format.

Discrete multi-enzymatic assemblies are visualised as a reaction pathway with arrows indicating reactions between intermediate products. Drawings are encoded in SVG format (Fig. 3B). For RiPPs, a simplified SVG ‘marble representation’ (Fig. 4B) can be created, where only the core peptide is depicted in full chemical detail and the leader and follower peptides are represented by a chain of amino acid ‘marbles’.

### AntiSMASH loading and module order detection

In order to process antiSMASH output of modular BGCs into RAIChU input, we added a basic module order detector to RAIChU’s antiSMASH module. First, all genes and antiSMASH-detected domains are parsed from an antiSMASH GenBank file. Then, based on strand direction, collinear gene blocks are extracted, and modules are predicted on each collinear gene block. If multiple collinear blocks exist in a BGC, they are rearranged in different orders until an order that minimises the number of broken modules has been found. Broken modules are modules that lack one or more domains that are required for chain elongation (Table 1).

For each combination, modules are predicted by iterating over all domains in order. This is done according to the following ruleset:

1. For the first domain in the sequence, start a new module.
2. If the module type of the current domain is not compatible with the module type of the previous domain, start a new module.
3. If a synthesis domain is encountered, start a new module, except when the current module only contains a single domain of unknown type that sits on the same gene. In that case, append the synthesis domain to the current module. This exception ensures that N-terminal docking domains are appended to the correct module.
4. If a TE or TD domain is encountered, append the domain to the previous module and end it. Standalone TE/TD domains are considered as a new module if there already exists another TE/TD domain in any previous module.
5. If a domain’s type already occurs in the current module, start a new module if there is a carrier domain in the current module, or if the domain occurs on a new gene with respect to the previous domain. Otherwise, append the domain to the current module.
6. If a domain has an unknown type, append it to the current module unless it starts on a new gene. If the domain is the only domain on its gene, store the domain as a separate module.
7. Otherwise, add the domain to the current module.

As the time complexity of order optimization is O(n!), with n the number of collinear blocks, we employed various approaches to limit the number of explored combinations. First, collinear blocks that begin with a module that is likely to function as a starter module, for instance because they contain a C-starter domain or do not contain synthesis domains, are prioritised as candidates for the first block in the sequence. Similarly, collinear blocks that end in a termination domain are prioritised as last blocks. Also, if there are more than 5 collinear blocks, any blocks that only contain a single domain are removed. Once an optimal combination has been chosen, any removed standalone domains and broken modules are moved to their initial locations.

### Validation and speed assessment

Drawing correctness and readability were assessed by randomly generating 1000 each of NRPS, *cis-*AT PKS, *trans-AT* PKS, all PKS, and hybrid NRPS/PKS biosynthetic gene clusters, generating 5000 clusters in total. Clusters were generated such that every cluster starts with an NRPS or PKS starter module, ends with an NRPS or PKS termination module, and contains anywhere between zero and eleven NRPS or PKS elongation modules. Facultative enzymatic domains and unspecified domains were randomly added to modules of their corresponding type, with the frequency of occurrence dependent on the selected substrate (NRPS) and other facultative enzymatic domains (PKS and NRPS) in the module. Substrate specificities were randomly selected from substrate subsets depending on the module type: the full set of 276 possible amino acid, β-amino acid and carboxylic acid substrates for NRPS starter modules; 196 amino acid and β-amino acid substrates for NRPS elongation and terminator modules; 24 CoA-tethered starter substrates for PKS starter modules; and 5 malonyl-CoA derivatives for PKS elongation and termination modules. Drawing correctness and readability were manually and computationally assessed for each of the 5000 randomly generated clusters, assessing three characteristics: overlaps (manual), central chain (60° or -60°; computational) and side chain (0° or 180°; manual) angles, and correctness of stereocentres (manual).

To measure RAIChU’s speed, we randomly generated BGCs of 1–10 modules long for NRPS, *cis-*AT PKS, *trans-AT* PKS, all PKS, and hybrid NRPS/PKS systems, generating 10 BGCs for each module-BGC-type combination to obtain mean and standard deviation. Speed was measured with the built-in Python ‘time’ module on a laptop running MacOS (Apple M1 chip; one core).

For validation against characterised biosynthetic products, we ran RAIChU on the entire MIBiG database [36] to catch general processing errors. From MIBiG, we also selected 10 NRPS, 10 PKS, and 10 NRPS/PKS hybrid clusters for manual curation. We collected SMILES strings directly from the MIBiG database where possible, and otherwise retrieved them from PubChem [44] or NP Atlas [45] in that order of preference. We also downloaded antiSMASH-generated GenBank files from the MIBiG database, loaded them into RAIChU, and added and changed substrate specificities of recognition domains and subtypes of KR domains to match experimentally verified KR domain subtypes and substrate specificities. We manually compared the resulting spaghetti diagrams with biosynthetic diagrams published in research articles for any inconsistencies and compared RAIChU’s rendered end products with the characterised natural products visualised by PIKAChU from SMILES strings. For our figures, we chose the predicted product that best matched the cyclisation site found in the characterised natural product.

We considered using fingerprinting methods such as Morgan fingerprinting for automatic validation of MIBiG BGCs. However, fingerprinting methods are sensitive to small modifications, particularly cyclisations. As RAIChU can generate natural product scaffolds but not mature natural products from raw antiSMASH output, predicted and real-world structures may not have a high fingerprinting similarity even when they are biosynthetically highly correlated. For this reason, we chose to manually curate a few select examples.

## Supplementary Information

Additional file 1

## Data Availability

The RAIChU software is made available under an open-source (MIT) licence and can be found at https://github.com/BTheDragonMaster/RAIChU and https://pypi.org/project/raichu/. A wiki can be found at https://github.com/BTheDragonMaster/RAIChU/wiki.

## Abbreviations

A: Adenylation domain
ACP: Acyl carrier protein
AT: Acyltransferase domain
BGC: Biosynthetic gene cluster
C: Condensation domain
Cyc: Heterocyclisation domain
DH: Dehydratase domain
E: Epimerization domain
ER: Enoylreductase domain
KR: Ketoreductase domain
NRP: Nonribosomal peptide
NRPS: Nonribosomal peptide synthetase
Ox: Oxidation domain
PCP: Peptidyl carrier protein
PKS: Polyketide synthase
RAIChU: Reaction Analysis through Illustrating Chemical Units
RiPP: Ribosomally synthesised and posttranslationally modified peptide
SMILES: Simplified Molecular-Input Line-Entry System
TC: Terpene cyclase
TD: Terminal reductase domain
TE: Thioesterase domain
antiSMASH: Antibiotics and Secondary Metabolite Analysis SHell
nMT: N-methylation domain
