# Supplementary material for: RAIChU: automating the visualisation of natural product biosynthesis
Source: J Cheminform. 2024 Sep 3;16:106. doi: 10.1186/s13321-024-00898-x (PMC11373092; doi:10.1186/s13321-024-00898-x)
Supplement: Supplementary file 1 — Additional file 1 [file 13321_2024_898_MOESM1_ESM.docx]

**RAIChU: automating the visualisation of natural product biosynthesis**

Additional File 1

Barbara R. Terlouw*^1^, Friederike Biermann*^1,2,3^, Sophie P.J.M. Vromans*^1^, Eric J. N. Helfrich^2,3#^, Marnix H. Medema^1#^

[1] Bioinformatics Group, Wageningen University, Droevendaalsesteeg 1, 6708 PB Wageningen,The Netherlands

[2] Institute for Molecular Bio Science, Goethe University Frankfurt, Max-von-Laue Strasse 9, 60438 Frankfurt am Main, Germany
[3] LOEWE Center for Translational Biodiversity Genomics (TBG), Senckenberganlage 25, 60325 Frankfurt am Main, Germany

* These authors contributed equally to this work

# Corresponding authors: [eric.helfrich@bio.uni-frankfurt.de](mailto:eric.helfrich@bio.uni-frankfurt.de) / [marnix.medema@wur.nl](mailto:marnix.medema@wur.nl)

Table of contents

| **Figure S1** | Reactions catalysed by core domains and common tailoring domains encoded in textbook PKS and NRPS biosynthetic gene clusters, which have been implemented into RAIChU. | S2 |
| --- | --- | --- |
| **Figure S2** | RAIChU’s implementation of six different KR subtypes, each leading to different stereochemical conformations. | S3 |
| **Figure S3** | RAIChU-rendered spaghetti diagram (A) and predicted products (B) of a 4-module hybrid NRPS/PKS cluster. | S3 |
| **Figure S4** | Options for scripting RAIChU that facilitate visualisation of multiple BGCs at once. | S4 |
| **Figure S5** | Overview of drawing readability issues | S5 |
| **Figure S6** | Validation on real cis-AT PKS biosynthetic gene clusters | S6 |
| **Figure S7** | Validation on real trans-AT PKS biosynthetic gene clusters | S7 |
| **Figure S8** | Validation on real NRPS biosynthetic gene clusters | S8 |
| **Figure S9** | Validation on real NRPS-PKS hybrid biosynthetic gene clusters | S9 |
| **Table S1** | Tailoring reactions that can be performed on the scaffold using RAIChU, their target atoms and required substrate(s). | S9 |
| **Table S2** | Example input table for the erythromycin BGC depicted in Figure 8.2B. | S13 |
| **Table S3** | BGCs from the MIBiG database used for validation. | S14 |
| **Table S4** | Drawing correctness and readability | S17 |
| **Table S5** | Drawing readability issues | S17 |
| **Supplementary discussion 1** | Background of natural product chemistry | S17 |
| 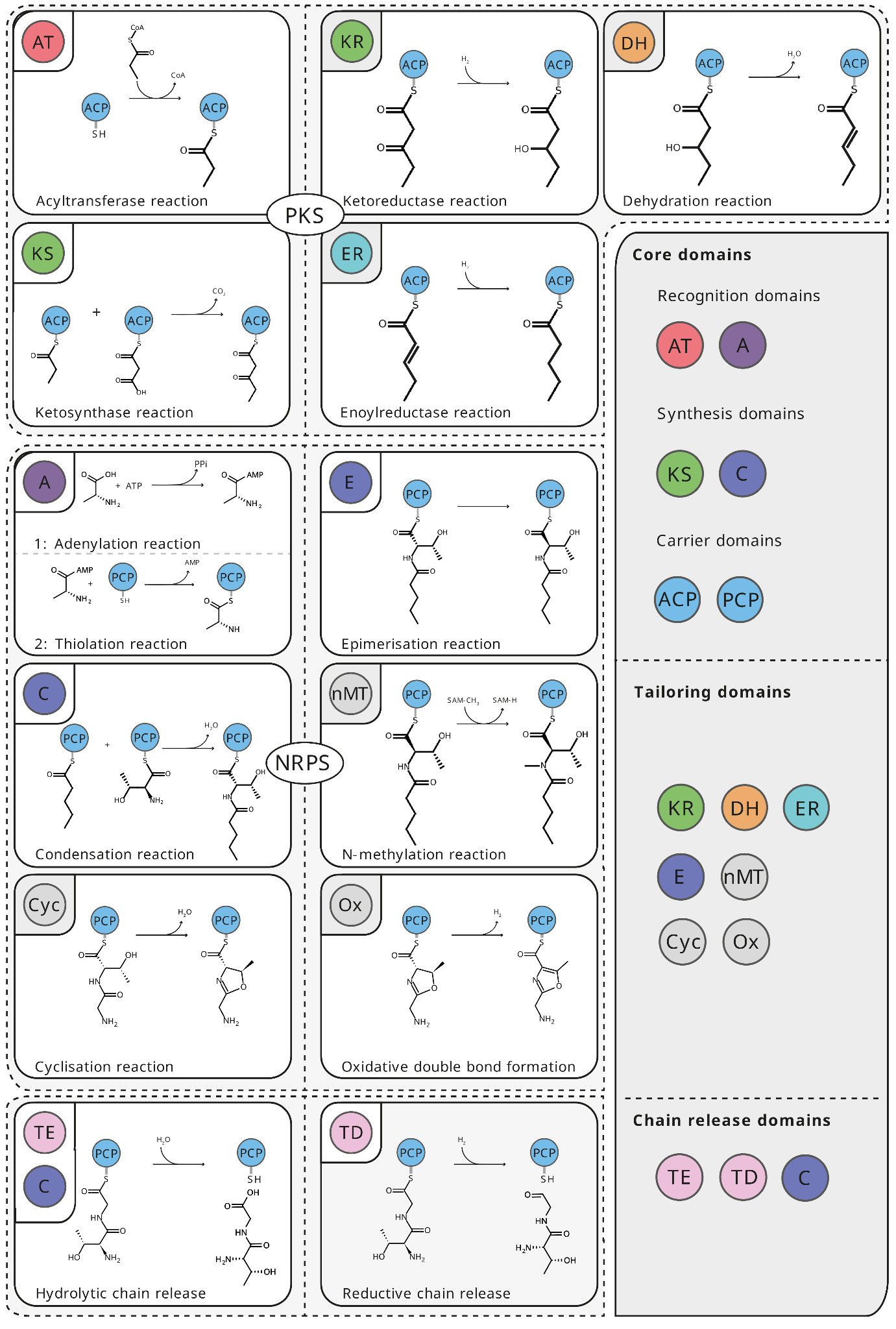 | |  |

***Figure S1. Reactions catalysed by core domains and common tailoring domains encoded in textbook PKS and NRPS biosynthetic gene clusters, which have been implemented into RAIChU.*** *Carrier domains (light blue) are not catalytic, but function as tethers to which NRP and polyketide intermediates are covalently linked.*


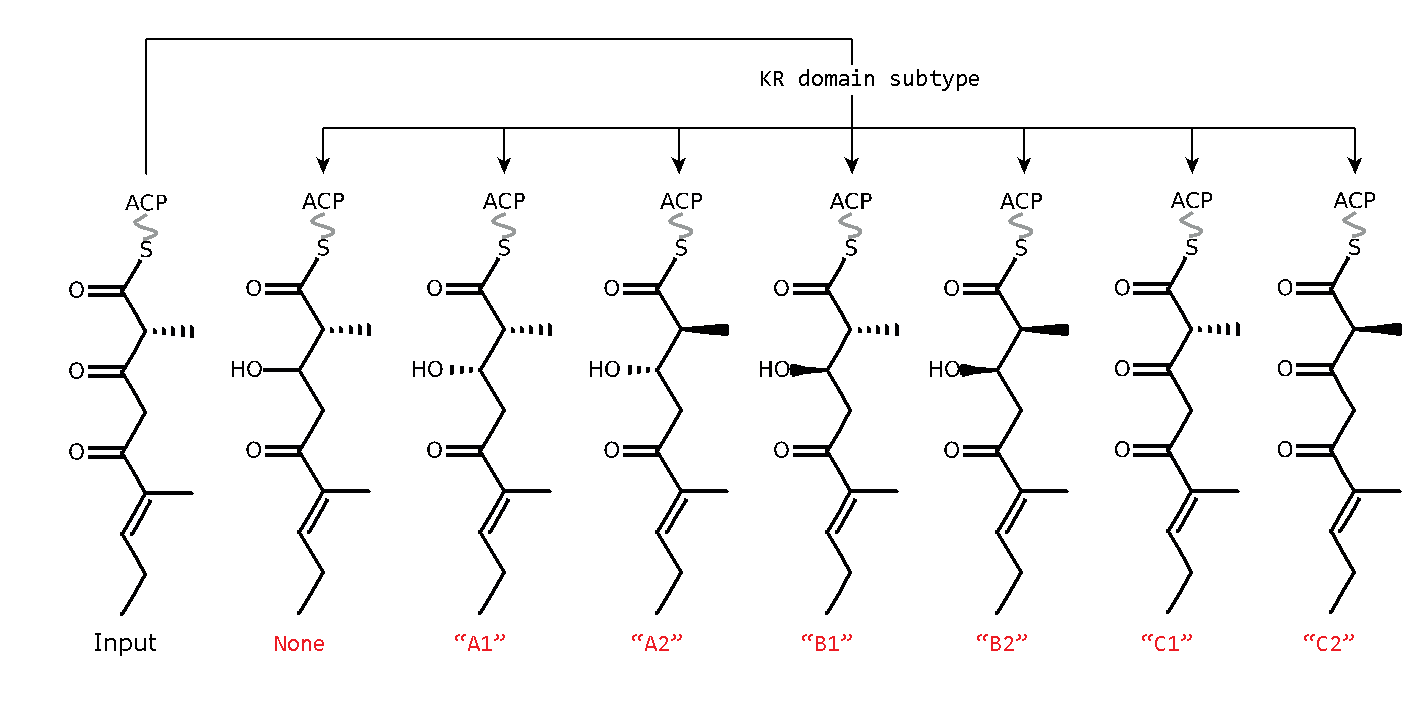


***Figure S2. RAIChU’s implementation of six different KR subtypes, each leading to different stereochemical conformations.***


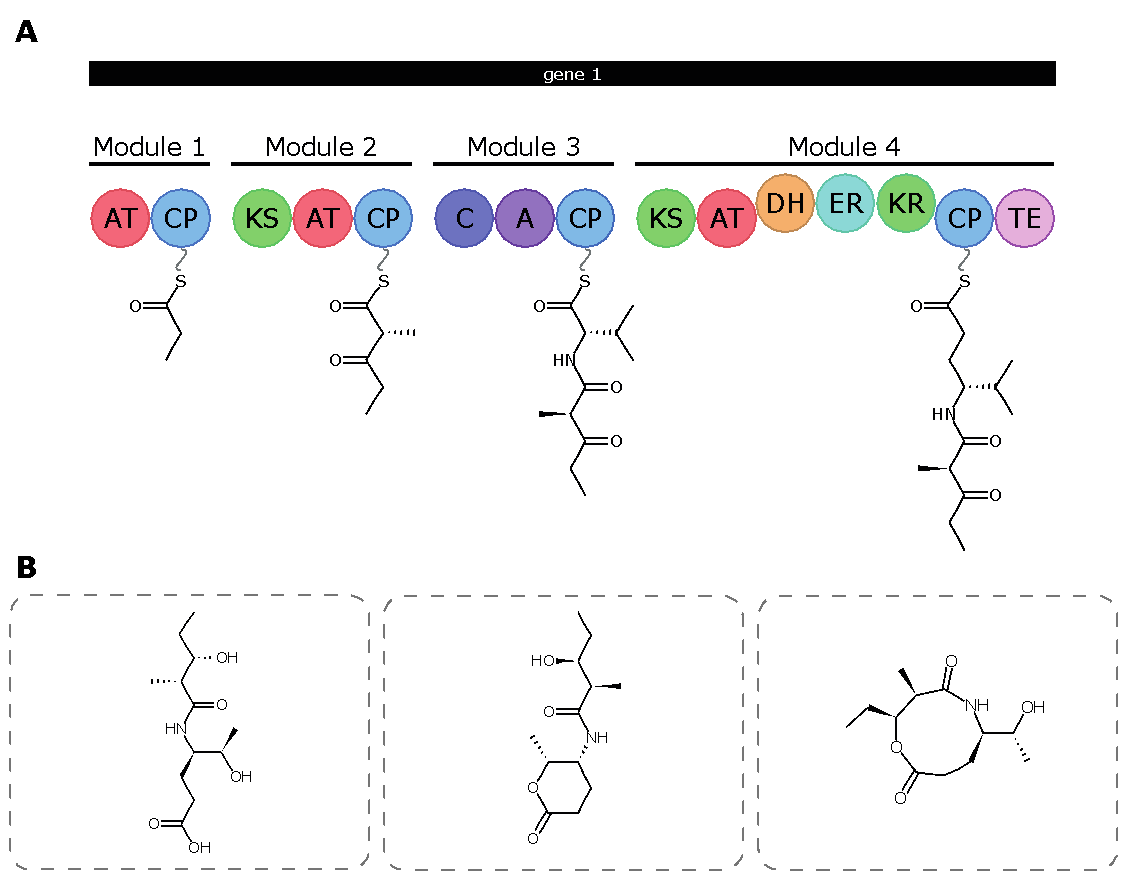


***Figure S3. RAIChU-rendered spaghetti diagram (A) and predicted products (B) of a 4-module hybrid NRPS/PKS cluster.***


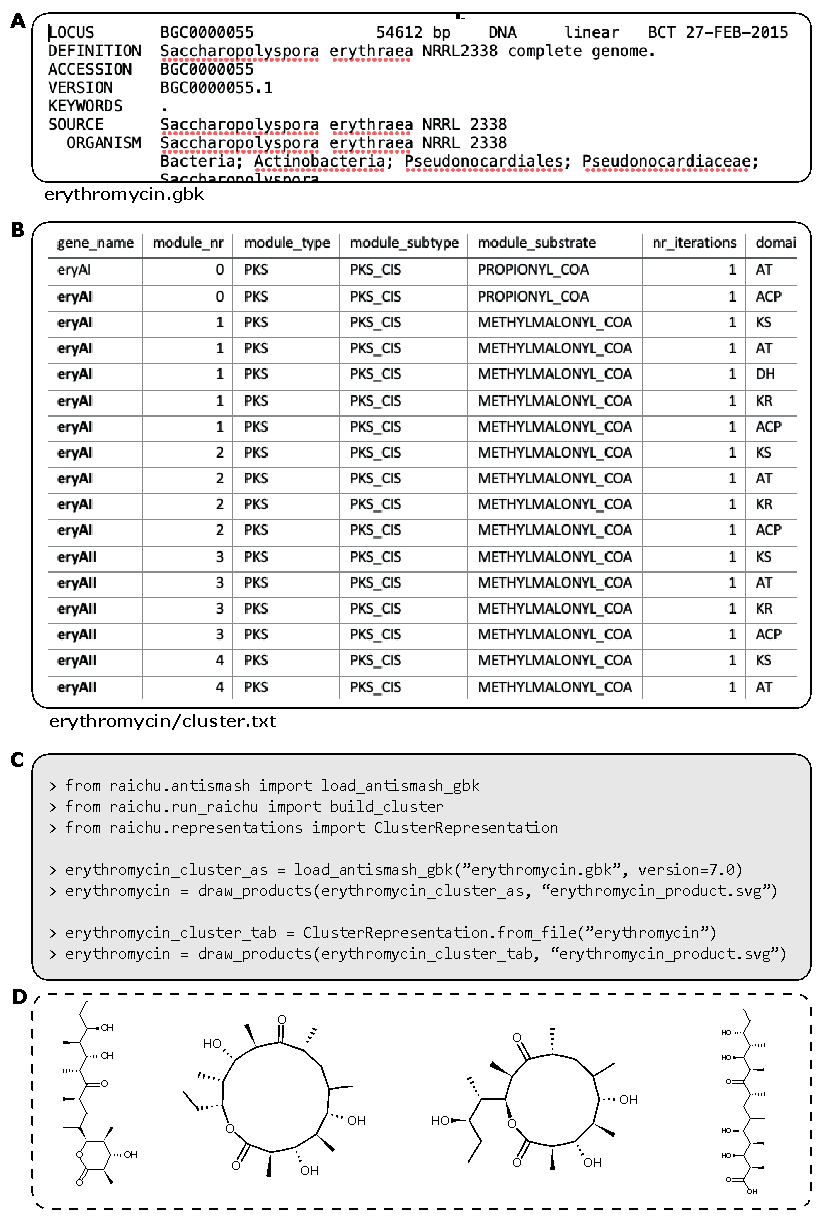


***Figure S4. Options for scripting RAIChU that facilitate visualisation of multiple BGCs at once.*** *From two different input formats (A, B), RAIChU can generate cluster and product visualisations with a single line of code (C). Resulting output products for the erythromycin BGC are shown in D. Note that the tabular format in B is missing some columns due to space constraints. For a comprehensive example, please refer to Table S2.*

*
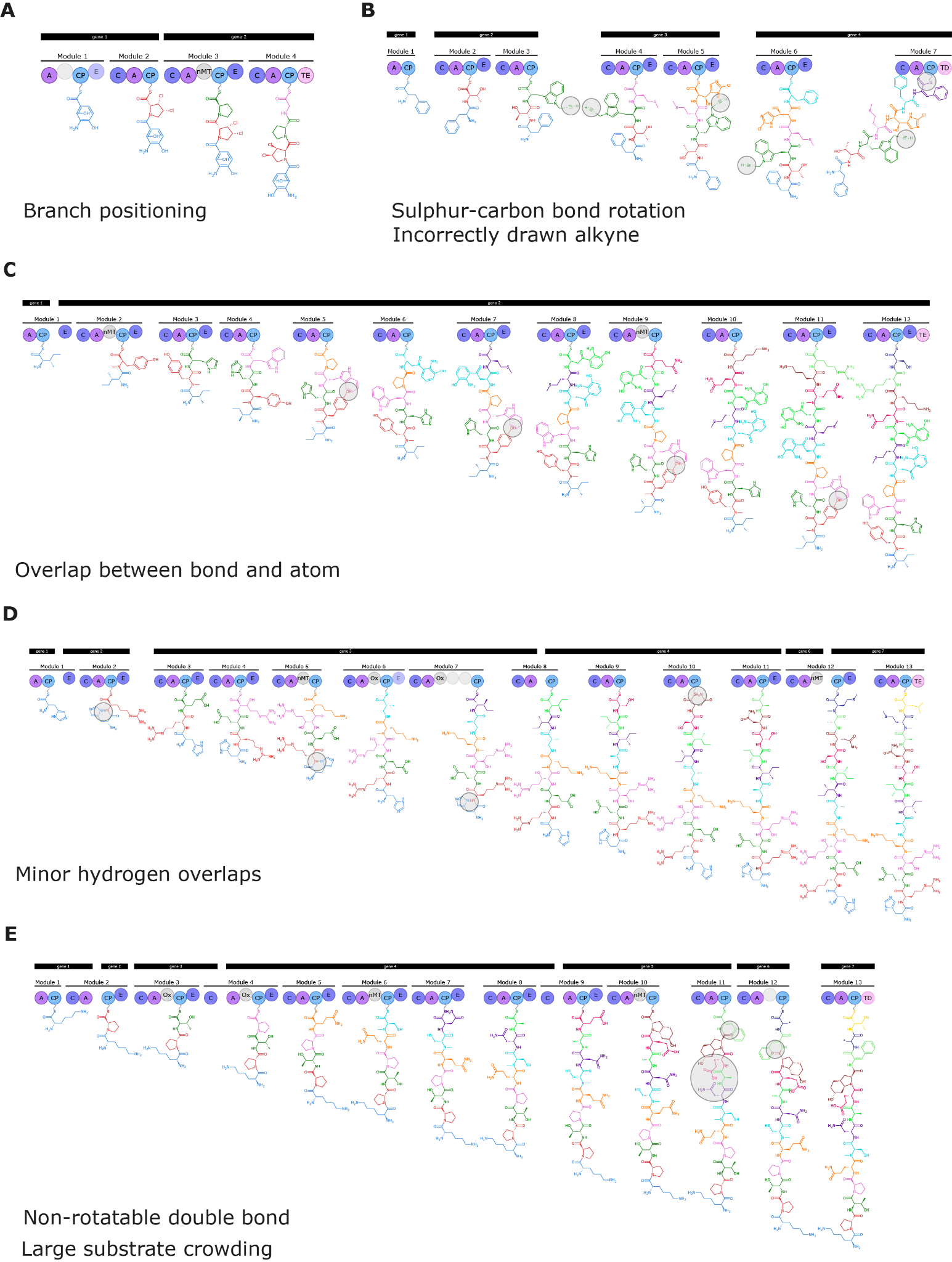
*

***Figure S5. Overview of drawing readability issues.*** *A. Branches adjacent to the central chain are sometimes positioned wrongly. B. The chain can be wrongly rotated when a substrate with a bulky, dehydrated side-chain is incorporated, and alkynes are drawn incorrectly. C. Atoms sometimes overlap with bonds. D. Minor overlaps can occur if two atoms that are close together in the plane both have hydrogen neighbours. E. Overlaps can occur due to double bonds not being considered rotatable, or due to unlimited space being available to draw a building block because of nearby large side chains.*

***
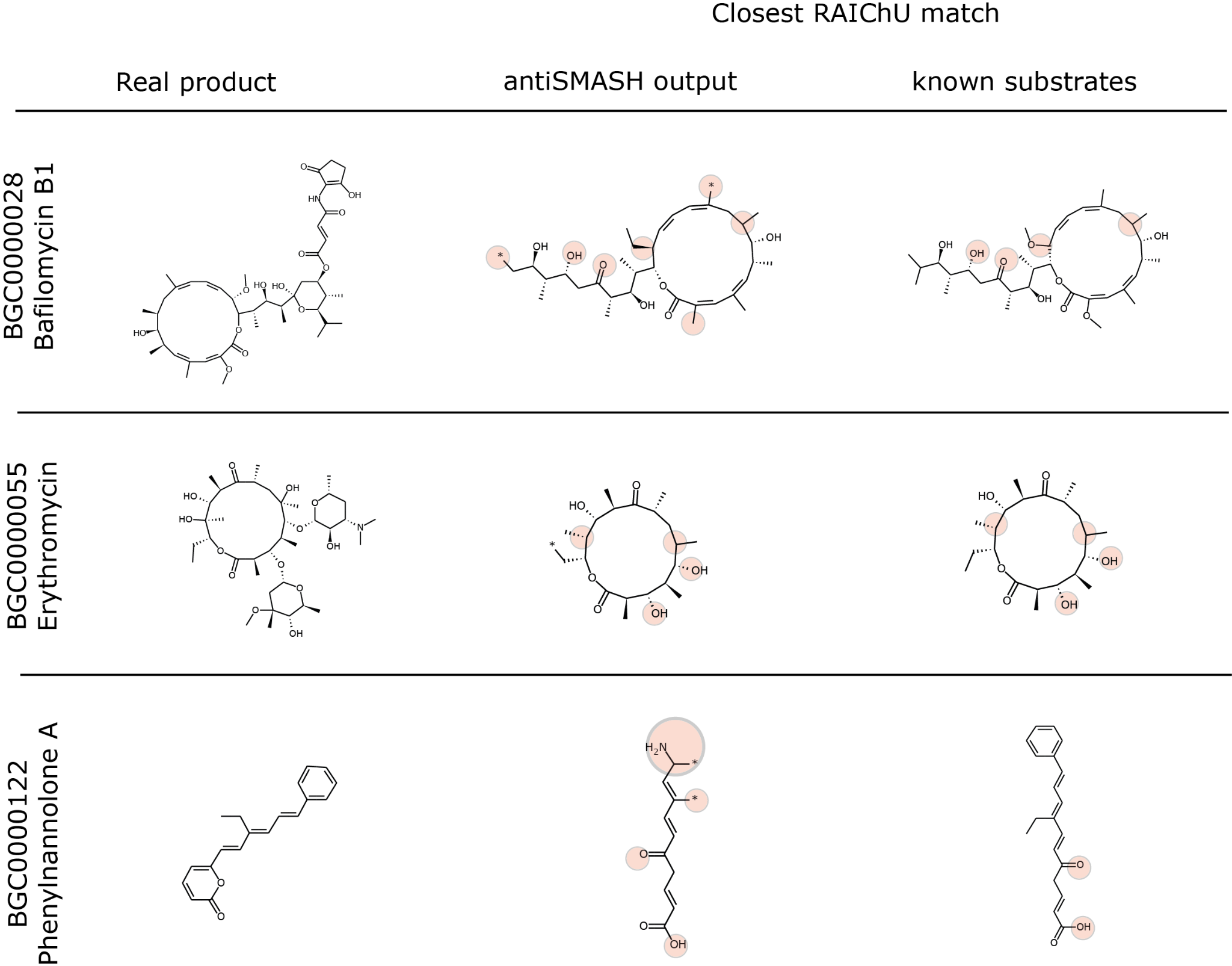
***

***Figure S6. Validation on real* cis-*AT PKS biosynthetic gene clusters.*** *Mistakes in all three examples stem from tailoring reactions such as cyclisations, glycosylations, and hydroxylations which are currently not possible to predict.*

***
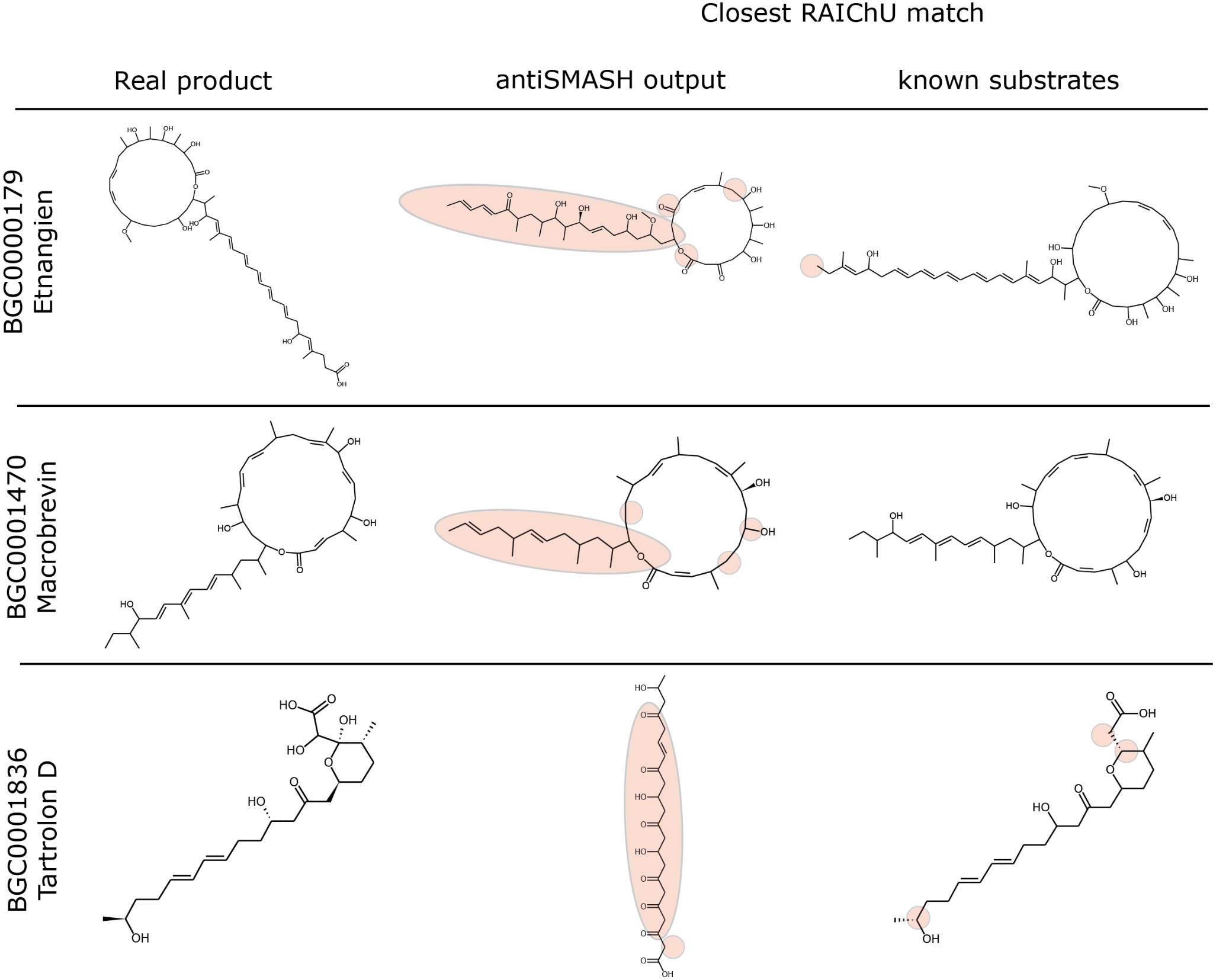
Figure S7. Validation on real* trans-*AT PKS biosynthetic gene clusters.*** *Mistakes in products predicted from raw antiSMASH output stem from the difficulty of correctly predicting trans-AT PKS chemistry from the KS domain.*

***
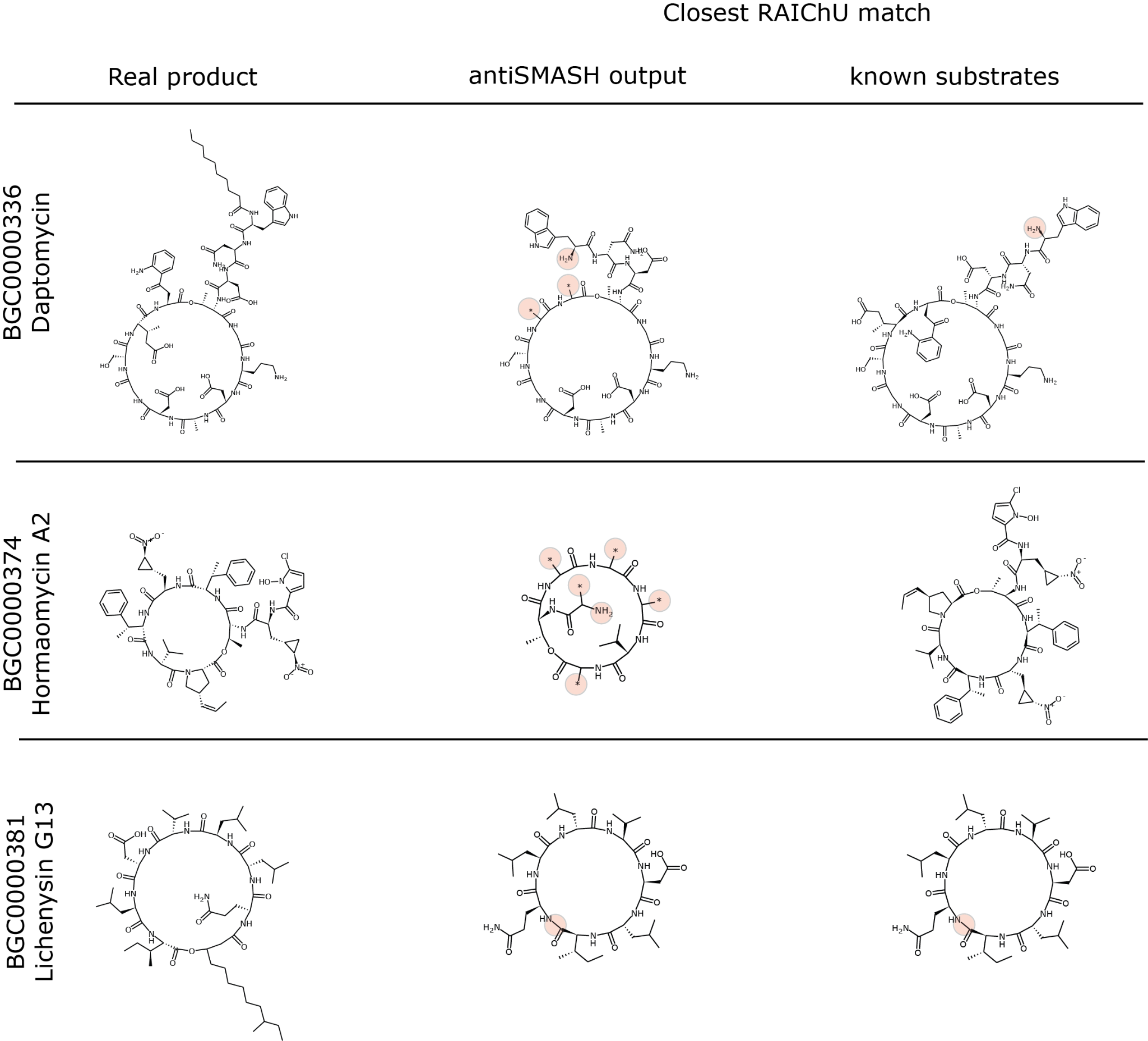
***

***Figure S8. Validation on real* NRPS *biosynthetic gene clusters.*** *Most mistakes in products predicted from raw antiSMASH output stem from incorrect substrate predictions. Mistakes in predicted products generated from edited antiSMASH output come from the presence of fatty acid starter substrates, which have not been implemented in RAIChU.*

***
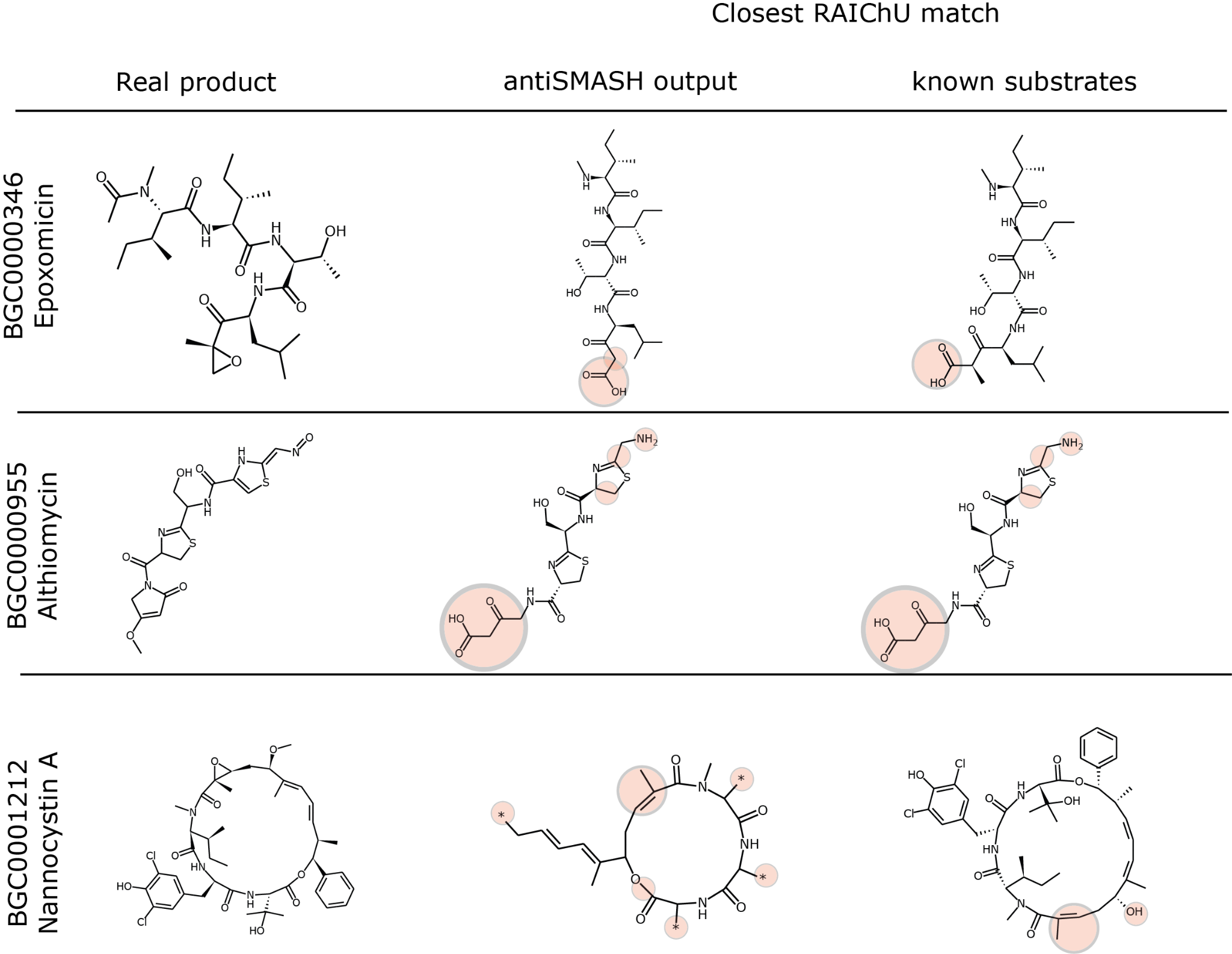
Figure S9. Validation on real NRPS-PKS hybrid biosynthetic gene clusters.*** *Mistakes stem from tailoring reactions such as cyclisations and hydroxylations which are currently not possible to predict.*

***Table S1. Tailoring reactions that can be performed on the scaffold using RAIChU, their target atoms and required substrate(s).*** *Atoms must always be given in the order given below.*

| **Tailoring Enzyme** | **Performed reaction** | **Required atoms** | **Substrate** |
| --- | --- | --- | --- |
| **Group transfer** | | | |
| METHYLTRANSFERASE | Methylation | Atom to add methyl group to | None |
| C_METHYLTRANSFERASE | Methylation | Carbon atom to add methyl group to | None |
| N_METHYLTRANSFERASE | Methylation | Nitrogen atom to add methyl group to | None |
| O_METHYLTRANSFERASE | Methylation | Oxygen atom to add methyl group to | None |
| HYDROXYLASE | Hydroxylation | Atom to add hydroxy group to | None |
| EPOXIDASE | Epoxidation of a double bond | Both atoms of the double bond to be epoxidised | None |
| PRENYLTRANSFERASE | Prenylation | Atom to add prenyl group to | Name of prenyl group ("DIMETHYLALLYL", "3_METHYL_1_BUTENYL", "GERANYL", "FARNESYL", "GERANYLGERANYL", "SQUALENE" OR "PHYTOENE") |
| ACETYLTRANSFERASE | Acetylation | Atom to add acetyl group to | None |
| ACYLTRANSFERASE | Acylation | Atom to add acyl group to | SMILES string of the substrate to add, with the first atom being the one to be directly bound to the natural product (e.g. C(=O)CCCCCCCC\C=C/CC) |
| AMINOTRANSFERASE | Transamination of a keto group | Oxygen of the keto group | None |
| HALOGENASE | Halogenation | Atom to add halogen to | Halogen atom |
| **Oxidoreduction** | | | |
| DOUBLE_BOND_REDUCTASE | Reduction of double bond | Both atoms of the double bond that is reduced | None |
| DOUBLE_BOND_ISOMERASE | Isomerization of double bond | Both atoms of the double bond to be shifted  Both atoms of the new double bond | None |
| DEHYDROGENASE | Formation of double bond | Both atoms of the bond that is oxidised | None |
| KETO_REDUCTASE | Reduction of keto group | Oxygen of the keto group | None |
| ALCOHOL_DEHYDROGENASE | Oxidation of hydroxy group to keto group | Oxygen of the hydroxy group | None |
| **Elimination** | | | |
| PEPTIDASE | Peptide bond cleavage | Carbon and nitrogen atom of the peptide bond to be cleaved | None |
| PROTEASE | Protein cleavage | Carbon and nitrogen atom of the peptide bond to be cleaved | None |
| MONOAMINE_OXIDASE | Deamination | Nitrogen of the amine group to be removed | None |
| DEHYDRATASE | Dehydration | Carbon with hydroxy group  Carbon to remove hydrogen from. Must be adjacent to first carbon | None |
| THREONINE_SERINE_DEHYDRATASE | Dehydration of threonine or serine | Oxygen atom of the threonine/serine | None |
| DECARBOXYLASE | Decarboxylation | Carbon atom of the carboxyl group to be removed | None |
| SPLICEASE | Removal of a part of the structure between two atoms (as seen in spliceotides) | Atoms flanking the substructure to be removed (input atoms are not removed) | None |
| ARGINASE | Arginine cleavage to L-ornithine | Secondary nitrogen of arginine | None |
| **Cyclisation** | | | |
| OXIDATIVE_BOND_SYNTHASE | Oxidative bond formation | Atoms to form bond between (each needs to neighbour at least one hydrogen) | None |
| MACROLACTAM_SYNTHETASE | Macrolactam formation between carboxy group and N-terminus | Hydroxy oxygen atom of the carboxy group | None |
| CYCLODEHYDRASE | Cyclodehydration of serine, threonine, or cysteine | Sulphur or oxygen atom of the sidechain in serine, threonine, or cysteine | None |
| LANTHIPEPTIDE_CYCLASE | Lanthipeptide cyclization | Sulphur of cysteine or β-carbon of dehydrated serine/threonine (for carbon bridge)  β-carbon of dehydrated serine/threonine | None |
| LANTHIONINE_SYNTHETASE | Lanthionine formation | Sulphur of cysteine or β-carbon of serine/threonine (for carbon bridge)  β-carbon of serine/threonine | None |
| THIOPEPTIDE_CYCLASE | Formation of nitrogen-containing six-membered ring in thiopeptides | β-carbon of dehydrated serine/threonine for which the upstream amino acid **is not** used in the 6 membered ring  β-carbon of dehydrated serine/threonine for which the upstream amino acid **is** used in the 6 membered ring | None |
| **Epimerisation** | | | |
| AMINO_ACID_EPIMERASE | Amino acid epimerization | α-carbon of the amino acid to be epimerised | None |
| **Bond breakage** | | | |
| HYDROLASE | Hydrolysis | Hetero atom, carbon atom of the bond to hydrolyse | None |
| REDUCTIVE_LYASE | Reductive single bond breakage | Atoms of the bond to break | None |

***Table S2. Example input table for the erythromycin BGC depicted in Figure Figure 8.2B.***

| gene_name | module_nr | module_type | module_subtype | module_substrate | nr_iterations | domain_type | domain_subtype | domain_name | domain_used | domain_active |
| --- | --- | --- | --- | --- | --- | --- | --- | --- | --- | --- |
| eryAI | 0 | PKS | PKS_CIS | PROPIONYL_COA | 1 | AT | None | AT | TRUE | TRUE |
| eryAI | 0 | PKS | PKS_CIS | PROPIONYL_COA | 1 | ACP | None | ACP | TRUE | TRUE |
| eryAI | 1 | PKS | PKS_CIS | METHYLMALONYL_COA | 1 | KS | None | KS | TRUE | TRUE |
| eryAI | 1 | PKS | PKS_CIS | METHYLMALONYL_COA | 1 | AT | None | AT | TRUE | TRUE |
| eryAI | 1 | PKS | PKS_CIS | METHYLMALONYL_COA | 1 | DH | None | DH | FALSE | FALSE |
| eryAI | 1 | PKS | PKS_CIS | METHYLMALONYL_COA | 1 | KR | B2 | KR | TRUE | TRUE |
| eryAI | 1 | PKS | PKS_CIS | METHYLMALONYL_COA | 1 | ACP | None | ACP | TRUE | TRUE |
| eryAI | 2 | PKS | PKS_CIS | METHYLMALONYL_COA | 1 | KS | None | KS | TRUE | TRUE |
| eryAI | 2 | PKS | PKS_CIS | METHYLMALONYL_COA | 1 | AT | None | AT | TRUE | TRUE |
| eryAI | 2 | PKS | PKS_CIS | METHYLMALONYL_COA | 1 | KR | A1 | KR | TRUE | TRUE |
| eryAI | 2 | PKS | PKS_CIS | METHYLMALONYL_COA | 1 | ACP | None | ACP | TRUE | TRUE |
| eryAII | 3 | PKS | PKS_CIS | METHYLMALONYL_COA | 1 | KS | None | KS | TRUE | TRUE |
| eryAII | 3 | PKS | PKS_CIS | METHYLMALONYL_COA | 1 | AT | None | AT | TRUE | TRUE |
| eryAII | 3 | PKS | PKS_CIS | METHYLMALONYL_COA | 1 | KR | C2 | KR | TRUE | TRUE |
| eryAII | 3 | PKS | PKS_CIS | METHYLMALONYL_COA | 1 | ACP | None | ACP | TRUE | TRUE |
| eryAII | 4 | PKS | PKS_CIS | METHYLMALONYL_COA | 1 | KS | None | KS | TRUE | TRUE |
| eryAII | 4 | PKS | PKS_CIS | METHYLMALONYL_COA | 1 | AT | None | AT | TRUE | TRUE |
| eryAII | 4 | PKS | PKS_CIS | METHYLMALONYL_COA | 1 | DH | None | DH | TRUE | TRUE |
| eryAII | 4 | PKS | PKS_CIS | METHYLMALONYL_COA | 1 | ER | None | ER | TRUE | TRUE |
| eryAII | 4 | PKS | PKS_CIS | METHYLMALONYL_COA | 1 | KR | None | KR | TRUE | TRUE |
| eryAII | 4 | PKS | PKS_CIS | METHYLMALONYL_COA | 1 | ACP | None | ACP | TRUE | TRUE |
| eryAIII | 5 | PKS | PKS_CIS | METHYLMALONYL_COA | 1 | KS | None | KS | TRUE | TRUE |
| eryAIII | 5 | PKS | PKS_CIS | METHYLMALONYL_COA | 1 | AT | None | AT | TRUE | TRUE |
| eryAIII | 5 | PKS | PKS_CIS | METHYLMALONYL_COA | 1 | KR | A1 | KR | TRUE | TRUE |
| eryAIII | 5 | PKS | PKS_CIS | METHYLMALONYL_COA | 1 | ACP | None | ACP | TRUE | TRUE |
| eryAIII | 6 | PKS | PKS_CIS | METHYLMALONYL_COA | 1 | KS | None | KS | TRUE | TRUE |
| eryAIII | 6 | PKS | PKS_CIS | METHYLMALONYL_COA | 1 | AT | None | AT | TRUE | TRUE |
| eryAIII | 6 | PKS | PKS_CIS | METHYLMALONYL_COA | 1 | KR | A1 | KR | TRUE | TRUE |
| eryAIII | 6 | PKS | PKS_CIS | METHYLMALONYL_COA | 1 | ACP | None | ACP | TRUE | TRUE |
| eryAIII | 6 | PKS | PKS_CIS | METHYLMALONYL_COA | 1 | TE | None | TE | TRUE | TRUE |

***Table S3. BGCs from the MIBiG database used for validation.***

| **MIBiG BGC identifier** | **BGC type** | **Product** | **DOI** |
| --- | --- | --- | --- |
| BGC0000028 | Cis - AT PKS type I | Bafilomycin B1 | 10.1002/cbic.201200743 |
| BGC0000038 | Cis - AT PKS type I | Coelimycin P1 | 10.1039/C2SC20410J |
| BGC0000055 | Cis - AT PKS type I | Erythromycin | 10.1021/cr9600316 |
| BGC0000072 | Cis - AT PKS type I | Gulmirecin A | 10.1002/chem.201404291 |
| BGC0000122 | Cis - AT PKS type I | Phenylnannolone A | 10.1002/cbic.201300676 |
| BGC0000144 | Cis - AT PKS type I | Salinomycin | 10.1128/AEM.06701-11 |
| BGC0000159 | Cis - AT PKS type I | Tautomycin | 10.1074/jbc.M804279200 |
| BGC0000165 | Cis - AT PKS type I | Tiacumicin B | 10.1021/ja109445q |
| BGC0001119 | Cis - AT PKS type I | Divergolide A | 10.1016/j.gene.2014.04.052 |
| BGC0001856 | Cis - AT PKS type I | Caniferolide A | 10.1039/C8OB03115K |
| BGC0000179 | Trans - AT PKS type I | Etnangien | 10.1021/ja804194c |
| BGC0000180 | Trans - AT PKS type I | Legioliulin | 10.1002/cbic.201300373 |
| BGC0000182 | Trans - AT PKS type I | Pseudomonic acid | 10.1021/ja501731p |
| BGC0000184 | Trans - AT PKS type I | Sorangicin A | 10.1002/cbic.201000313 |
| BGC0001470 | Trans - AT PKS type I | Macrobrevin | 10.1038/s41564-018-0200-0 |
| BGC0001836 | Trans - AT PKS type I | Tartrolon D | 10.1073/pnas.1213892110 |
| BGC0002060 | Trans - AT PKS type I | Secimide | 10.1038/s41467-021-21163-x |
| BGC0000083 | Trans - AT PKS type I | Lactimidomycin | 10.1021/bi501396v |
| BGC0000336 | NRPS | Daptomycin | 10.1099/mic.0.27757-0 |
| BGC0000374 | NRPS | Hormaomycin | 10.1016/j.chembiol.2010.12.018 |
| BGC0000381 | NRPS | Lichenysin D | 10.1128/JB.181.1.133-140.1999 |
| BGC0000415 | NRPS | Quinomycin | 10.1371/journal.pone.0056772 |
| BGC0000440 | NRPS | Teicoplanin | 10.1099/mic.0.26507-0 |
| BGC0000447 | NRPS | Tolaasin I | 10.1002/cbic.201300553 |
| BGC0000449 | NRPS | Tridecaptin A1 | 10.1002/cbic.201300595 |
| BGC0000452 | NRPS | Tyrocidine | 10.1007/s11274-018-2437-4 |
| BGC0001192 | NRPS | Colistin A | 10.1007/s00203-015-1084-5 |
| BGC0001214 | NRPS | Marformycin A | 10.1021/acs.orglett.5b00389 |
| BGC0000346 | NRPS/PKS hybrid | Epoxomicin | 10.1021/cb400699p |
| BGC0000955 | NRPS/PKS hybrid | Althiomycin | 10.1002/cbic.201100154 |
| BGC0000963 | NRPS/PKS hybrid | Bleomycin | 10.1016/S1074-5521(00)00011-9 |
| BGC0001047 | NRPS/PKS hybrid | Syringolin A | 10.1094/MPMI.2004.17.1.90 |
| BGC0001052 | NRPS/PKS hybrid | Tirandamycin | 10.1002/cbic.200900658 |
| BGC0001165 | NRPS/PKS hybrid | Curacin A | 10.1021/np0499261 |
| BGC0001212 | NRPS/PKS hybrid | Nannocystin A | 10.1002/anie.201505069 |
| BGC0001230 | NRPS/PKS hybrid | Salinamide A | 10.1002/anie.201508576 |
| BGC0001331 | NRPS/PKS hybrid | BE-43547 | 10.1038/nchem.2657 |
| BGC0001342 | NRPS/PKS hybrid | CMC-Thuggacin | 10.1016/j.chembiol.2010.02.013 |

***Table S4. Drawing correctness and readability***

| **BGC type** | **Chemically correct drawings** | **Visually readable drawings** |
| --- | --- | --- |
| *cis*-AT PKS | 1000 (100%) | 1000 (100%) |
| *trans*-AT PKS | 1000 (100%) | 1000 (100%) |
| PKS | 1000 (100%) | 1000 (100%) |
| NRPS | 1000 (100%) | 906 (90.6%) |
| PKS-NRPS hybrid | 1000 (100%) | 977 (97.7%) |

***Table S5. Drawing readability issues.*** *An example of each readability issue is shown in Figure S6, Additional File 1. Note that multiple*

| **Readability issue** | **Occurrence in NRPS** | **Occurrence in NRPS-PKS hybrids** |
| --- | --- | --- |
| Minor hydrogen overlaps | 71 (7.1%) | 6 (0.6%) |
| Non-rotatable double bonds | 10 (1.0%) | 8 (0.8%) |
| Large substrate crowding | 6 (0.6%) | 9 (0.9%) |
| Alkyne drawing | 6 (0.6%) | 2 (0.2%) |
| Overlaps between bonds and atoms | 5 (0.5%) | 0 (0.0%) |
| Sulphur-carbon bond rotation | 5 (0.5%) | 2 (0.2%) |
| Branch positioning | 1 (0.1%) | 0 (0.0%) |

***Supplementary Discussion 1: Background of modular natural product chemistry***

For NRPS and PKS systems, respectively, carrier domains are called peptidyl carrier protein (PCP) domains and acyl carrier protein (ACP) domains, recognition domains are called adenylation (A) and acyltransferase (AT) domains, and condensation domains are called condensation (C) and ketosynthase (KS) domains [[16–18]](https://paperpile.com/c/4bcmlt/Q893+kK6q+vUZX).

In addition to these core domains, NRPS and PKS modules can also contain on-line tailoring domains that modify the incorporated building blocks. For NRPS modules, the most prevalent of these are epimerization (E) domains, which change the configuration of the α-carbon of amino acid building blocks, and *N*-methylation (nMT) domains, which methylate the α-amino group of amino acid subunits. Other frequently encountered module-encoded domains include heterocyclisation (Cyc) and oxidation (Ox) domains. These domains typically work sequentially, and catalyse heterocyclisation reactions between threonine, serine or cysteine residues and the amino acid backbone to form azoline rings, followed by oxidation to azole rings, respectively. In PKS enzymes, tailoring domains likewise act sequentially: the ketoreductase (KR) domain reduces the β-ketoacyl-S-ACP formed by the ketosynthase to a β-hydroxyacyl-S-ACP, the dehydratase (DH) domain forms a double bond by elimination of the β-hydroxy group and forming a α,β-enoyl-S-ACP intermediate, and the enoylreductase (ER) domain reduces this double bond, creating a saturated acyl-S-ACP. Terminal NRPS and PKS modules may additionally contain a thioesterase (TE), condensation or terminal reductase (TD) domain, which catalyse the release of the natural product from the enzyme, either as a linear free acid or a macrocyclic lactone/lactam [[17–19]](https://paperpile.com/c/4bcmlt/kK6q+vUZX+qh0l). The most commonly occurring PKS/NRPS domains and the reactions they catalyse are summarised in Figure S1.

The structure of a natural product scaffold is not only determined by domain and module composition, but also by the substrate specificity of the recognition domains, which select a building block from a wide variety of substrates each resulting in a scaffold with different properties. This observation is especially true for A domains, that are part of every NRPS module and which recognize as many as five hundred different substrates, including L- and D-amino acids, a wide variety of non-proteinogenic amino acids, and even fatty acids, aryl acids, and hydroxy acids, thus greatly exceeding the level of peptide sequence diversity achievable through ribosomal peptide synthesis [[21, 22]](https://paperpile.com/c/4bcmlt/6bw3+KZ6M). In contrast, the AT domains of PKS modules select a modest variety of acyl-CoA thioester substrates. In most cases, malonyl-CoA and its methylated variant methylmalonyl-CoA are used as building blocks [[23]](https://paperpile.com/c/4bcmlt/wwly). Software tools exist that predict the specificity of modular NRPS and PKS recognition domains from their protein sequence, such as the AT domain specificity predictor published by Minowa *et al.* [*[24]*](https://paperpile.com/c/4bcmlt/GA2C), the A domain substrate predictors AdenPredictor [[25]](https://paperpile.com/c/4bcmlt/Iesw), SANDPUMA [[26]](https://paperpile.com/c/4bcmlt/vOIf), NRPSPredictor2 [[27]](https://paperpile.com/c/4bcmlt/voFz) and AdenylPred [[28]](https://paperpile.com/c/4bcmlt/n7Qr), several of which have been incorporated into larger BGC detection and analysis tools such as antiSMASH [[29]](https://paperpile.com/c/4bcmlt/HuxD) and PRISM [[30]](https://paperpile.com/c/4bcmlt/vozp).
